# Preparation of a Mini-Library of Thermo-Responsive Star (NVCL/NVP-VAc) Polymers with Tailored Properties Using a Hexafunctional Xanthate RAFT Agent

**DOI:** 10.3390/polym10010020

**Published:** 2017-12-24

**Authors:** Norma Aidé Cortez-Lemus, Angel Licea-Claverie

**Affiliations:** Centro de Graduados e Investigación en Química, Instituto Tecnológico de Tijuana, A. P. 1166, Tijuana 22000, B. C., Mexico; aliceac@tectijuana.mx

**Keywords:** R-RAFT, star polymers, poly(*N*-vinylcaprolactam), poly(vinyl acetate), poly(*N*-vinylpyrrolidone), end-group functionality, micelles, aggregates, methotrexate

## Abstract

A mini-library of star-shaped thermoresponsive polymers having six arms was prepared using a hexafunctional xanthate by reversible addition–fragmentation chain transfer (RAFT) polymerization. Star polymers with homopolymeric arms of poly(*N*-vinylcaprolactam) (PNVCL), copolymeric arms of poly(*N*-vinylcaprolactam-*co*-*N*-vinylpyrrolidone) (PNVCL-*co*-PNVP) and also arms of block copolymers of PNVCL-*b*-PVAc, (PNVCL-*co*-PNVP)-*b*-PVAc, and combinations of them changing the order of the block was achieved exploiting the R-RAFT synthetic methodology (or R-group approach), wherein the thiocarbonyl group is transferred to the polymeric chain end. Taking advantage of the RAFT benefits, the molecular weight of the star polymers was controlled (M_n_ = 11,880–153,400 g/mol) to yield star polymers of different sizes and lower critical solution temperature (LCST) values. Removing the xanthate group of the star polymers allowed for the introduction of specific functional groups at the ends of the star arms and resulted in an increase of the LCST values. Star PNVCL-*b*-PVAc diblock copolymers with PVAc contents of 5–26 mol % were prepared; the hydrophobic segment (PVAc) is located at the end of the star arms. Interestingly, when the PVAc content was 5–7 mol %, the hydrodynamic diameter (D_h_) value of the aggregates formed in water was almost the same sa the D_h_ of the corresponding PNVCL star homopolymers. It is proposed that these star block copolymers self-assemble into single flowerlike micelles, showing great stability in aqueous solution. Star block copolymers with the PVAc hydrophobic block in the core of the star, such as PVAc-*b*-(PNVCL-*co*-PNVP), form micellar aggregates in aqueous solution with D_h_ values in the range from ~115 to 245 nm while maintaining a thermoresponsive behavior. Micellar aggregates of selected star polymers were used to encapsulate methotrexate (MTX) showing their potential in the temperature controlled release of this antineoplasic drug. The importance of the order in which each block constituent is introduced in the arms of the star polymers for their solution/aggregation behavior is demonstrated.

## 1. Introduction

Star-like polymers exhibit a polymer architecture that initially attracted attention due to its unique rheological properties [1]. In the last decade, the areas of research where the stars are the protagonists has diversified enormously [2]. Those studies focus on advanced applications such as drug delivery [3,4,5], nanoreactors for confined catalysis [6] and imaging [7,8]. There are several methods for the preparation of star polymers with controlled number of arms [9,10,11,12,13,14,15,16]. One of the most promising is the use of reversible addition–fragmentation chain transfer (RAFT) polymerization by the core-first approach [17,18,19,20]. In this methodology, a thio-group containing chain transfer agent, also called RAFT-agent, can be linked to the nucleus of the star (Z-group approach) or at the end of the arms of the star (R-group approach). In the last years, it has been proven that the R-group approach allows for a better control of the star polymer characteristics, since star to star coupling reactions are avoided [21,22]. The preparation of polymers using the R-group approach opens up a whole range of functionalization possibilities at the ends of the polymer chains through post-functionalization reactions [23,24]. For example, the thiol functionality allows the stabilization of gold nanoparticles, and preparation of biopolymers conjugates, block copolymers, nano-objects, etc. [25].

Polymer micelles prepared from amphiphilic block copolymers that can host poorly water-soluble drugs have been in the focus of interest for nanopharmaceuticals in the last twenty years [26,27,28,29,30]. Poly(*N*-vinylcaprolactam)-*b*-poly(vinyl acetate) (PNVCL-*b*-PVAc) is an interesting amphiphilic combination that has emerged in the last years. PNVCL is biocompatible, thermoresponsive and water-soluble below its lower critical solution temperature (LCST) [31,32,33,34,35,36]. For its part, PVAc is a hydrophobic, biocompatible and biodegradable polymer used in pharmaceutical applications [37,38,39,40,41,42,43,44,45,46].

There are several reports in the literature on the preparation of PNVCL-*b*-PVAc block copolymers [37,47,48,49,50,51,52]. Wan et al. [37] reports on the synthesis of PVAc-*b*-PNVCL copolymers by RAFT polymerization. PVAc was used as a macro-CTA for the polymerization of NVCL. However, the authors do not report studies on solution or thermosensitivity behavior of these copolymers. Detrembleur et al. [51,52] studied the thermoresponsive behavior of PVAc-*b*-PNVCL copolymers. Well defined block copolymers were obtained when the polymerization of NVCL was initiated using poly(vinyl acetate)-Co(acac)_2_ macroinitiator by cobalt-mediated radical polymerization. These copolymers displayed temperature transitions depending of the ratio of PVAc in the copolymer (26.5 to 29.5 °C).

The design of block copolymers focused on obtaining stable micellar systems in aqueous solution is one of the main objectives for their application in nanomedicine. One approach in the last years has been the use of different topologies of amphiphilic polymers to yield more stable micelles. One example is the use of star polymers [53,54]. To date, there are only a few studies in the literature on the preparation of star-shaped block copolymers based on PNVCL [32,33,34]; however, to the best of our knowledge, no study has reported on star copolymers based on PNVCL and PVAc as part of block copolymeric arms. 

In this report, the synthesis and characterization of a mini-library of star-shaped polymers containing PNVCL by using a hexafunctional xanthate chain transfer agent is described. Variations in the arms of the star polymers included: the introduction of the hydrophobic PVAc in the star core or at the end of the arms, the incorporation of the more hydrophilic NVP as comonomer with NVCL to change the LCST in the star core, and the preparation of blocks of (PNVCL-*co*-PNVP) in the star polymer core and at the end of the arms in combination with a PVAc block. Furthermore, the properties in aqueous solution of the star polymers were investigated, regarding its aggregation behavior and lower critical solution temperature (LCST). Selected star polymers were used to encapsulate methotrexate [55,56,57] (MTX) precluding it´s application in controlled release of drugs.

## 2. Experimental

### 2.1. Materials

Dipentaerythritol, (DPERT, 85%, Aldrich, St. Louis, MO, USA), *N-*vinylpyrrolidone (NVP, 99%, Aldrich), Vinyl acetate (VAc, >99%, Aldrich), stannous 2-ethylhexanoate (Sn(Oct)_2_, 99%, Aldrich), triethylamine (TEA, 99.5%), 4,4-azobis(4-cyanovaleric acid) (ACVA) (Fluka, St. Louis, MO, USA), 2,2′-azobis(4-methoxy-2,4-dimethyl valeronitrile) (V-70, Wako Chemicals, Richmond, VA, USA, 96%), 2-bromopropionyl bromide (Aldrich, 97%), methotrexate hydrate (MTX, Sigma-Aldrich, St. Louis, MO, USA, 98%), anhydrous sodium sulfate (Na_2_SO_4_), *N*,*N*-dimethylacetamide (Aldrich, 99.8%), *N*,*N*-dimethylformamide (DMF, Fermont, Monterrey, Mexico, 99.8%), tetrahydrofuran (THF, Fermont, 99.9%), *p*-dioxane (Sigma-Aldrich, 99.8%), dichloromethane (DCM, Fermont, Monterrey, Mexico, 99.5%), potassium ethyl xanthogenate (Aldrich, 97%), and hydrochloric acid (HCl, 10% aqueous solution, Fermont) were used as received. NVCL (Aldrich, >99%) was purified by recrystallization with hexane. Column chromatographic purifications were performed using silica gel (70–230 mesh, Acros Organics, Morris Plains, NJ, USA).

### 2.2. Measurements

Dynamic light scattering (DLS) measurements were carried out on 1.0 mg/mL star polymer solutions/dispersions at 20 or 25 °C using a Malvern Instruments Nano-ZS Nanosizer (ZEN 3690) equipment. The instrument is equipped with a helium neon laser (633 nm) with a size detection range of 0.6 nm–5 μm. DLS experiments were performed at the scattering angle of 90° and equilibrated for 10 min before data collection. The solutions/dispersions were filtered through a 0.45 μm nylon membrane filter before analysis to remove dust. The volume-average hydrodynamic diameter (D_h_) and polydispersity index (PDI) were calculated using Malvern Instruments dispersion technology software, based on CONTIN analysis and Stokes-Einstein equation for spheres as usual. The LCST was taken as the temperature at which the star polymer was still soluble (before the solution started to turn cloudy) at a 1 mg/mL concentration. The LCST was measured by DLS using the Nano-ZS Nanosizer equipment with a temperature program that increased from 20 to 50 °C in two degree steps, equilibrating for 4 min once the measurement temperature was achieved; measurements were performed three times, each of which included three 30 s runs. Gel permeation chromatography (GPC) was performed on a Varian 9002 chromatograph equipped with a series of three columns (Phenogel: OH-646-K0, OH-645-K0 and OH-643-K0) and two detectors: a refractive index detector (Varian RI-4) and a triangle light scattering detector (LS detector MINI-DAWN, Wyatt). The measurements were performed in THF at 35 °C. Polystyrene standards were used for calibration of the LS detector. THF was used as the mobile phase at a flow rate of 0.7 mL/min. Sample solutions were prepared using 20 mg/mL concentration and filtered through a 0.45 μm PTFE membrane filter before analysis. Images of the formed aggregates were acquired via atomic force microscopy (AFM) using an Agilent SPM 5100 Molecular Imaging instrument in tapping mode equipped with commercial silicon cantilevers and a high resolution scanner. Samples were dispersed in water and stirred for 48 h (*c* = 0.02 mg/mL), and then a drop of the dispersion was deposited onto a mica surface and allowed to evaporate.

^1^H and ^13^C NMR spectra were collected on a Bruker AMX-400 (400 MHz) spectrometer and are reported in ppm using TMS as internal standard. The solvent used was deuterated chloroform, CDCl_3_, for all samples. UV-vis spectra of the star-shaped PNVCL polymers and copolymers were recorded using a UV-Vis Varian Cary 100 Spectrophotometer at room temperature.

### 2.3. Synthetic Methods 

#### 2.3.1. Preparation of the Hexafunctional Chain Transfer Agent (CTA-1 or R-RAFT Agent) 

The synthesis of the CTA-1 was carried out according to the methodology reported by Stenzel et al. [40] but using dipentaerythritol (DPERT) for the core instead of pentaerythritol.

Hexafunctional bromide RAFT agent precursor. ^1^H NMR (CDCl_3_): 1.83 (C*H*_3_–C–Br, 18H, d, *J* = 4.0 Hz), 4.24–4.14 (C*H*–Br, 6H, m), 3.52 (C*H*_2_–O, 4H, s), 4.44–4.27 (C*H*_2_–O, 12H, m) (see Figure S1). ^13^C NMR (CDCl_3_): 21.57 (*C*H_3_), 39.54 (C–*C*–C), 43.95 (CH_3_*C*HBr), 63.21 (*C*H_2_–O), 69 (*C*H_2_–O–*C*H_2_), 169.51 (C=O). White solid (65%) (see Figure S2).

Hexafunctional RAFT agent (CTA-1 or R-RAFT agent). ^1^H NMR (CDCl_3_): 1.44–1.41 (C*H*_3_–CH_2_–O, 18H, t, *J* = 7.2 Hz), 1.58 (C*H*_3_–CH–S, 12H, d, *J* = 7.2 Hz), 3.41 (C*H*_2_–O, 4H, s), 4.19–4.09 (CH_3_C*H*_2_–O, 12H, m), 4.46–4.40 (CH_3_C*H*S, 6H, q, *J* = 7.6 and *J* = 7.2 Hz), 4.67–4.62 (C*H*_2_–O, 12H, q, *J* = 6.8 and *J* = 7.2 Hz) (see Figure S3). ^13^C NMR (CDCl_3_): 13.75 (*C*H_3_–CH_2_–O), 16.64 (*C*H_3_–C–S), 42.49 (C–*C*–C), 47.09 (*C*HS), 62.92 (*C*H_2_–O), 70.64 (CH_3_–*C*H_2_–O), 171.3 (C=O), 212.6 (C=S). Viscous yellowish liquid (45%) (see Figure S4).

#### 2.3.2. Preparation of Star (PNVCL) with Six Arms

The following procedure is typical for a target star polymer (PNVCL_34_)_6_: CTA (12 mg, 0.0093 mmol), NVCL (3 g, 21.58 mmol) and 2,2′-azobis(4-methoxy-2,4-dimethyl valeronitrile, V-70) (1 mg, 0.003 mmol) were dissolved in 1.5 mL of *p*-dioxane and mixed in a Schlenk flask containing a magnetic stir bar. The solution was de-oxygenated by bubbling nitrogen for 20 min at room temperature. Then, the flask was placed in an oil bath preheated at 30 °C. At designated time, the polymerization was stopped by cooling to room temperature. The polymerization yield was obtained gravimetrically by adding a three-fold excess of diethyl ether. The polymer was obtained as a white solid (18%), M_n GPC_ = 28,100 g/mol, Ð = 1.06 (Table 1, Entry 3).

^1^H NMR (CDCl_3_, TMS, δ, ppm): 4.75–4.17 (bs, –NC*H*), 3.5–2.89 (bs, –NC*H*_2_), 2.74–2.08 (bs, –COC*H*_2_), 2.03–1.02 (bm, NCH_2_C*H*_2_C*H*_2_C*H*_2_ of caprolactam ring and C*H*_2_ of backbone) (see Figure S5).

#### 2.3.3. Preparation of Star PNVCL-*b*-PVAc Block Copolymers with Six Arms

Star PNVCL-macroCTA (M_n GPC_ = 82,600 g/mol, Ð = 1.01, 0.27 g, 0.003 mmol), VAc (0.17 g, 1.97 mmol), and 4,4-azobis(4-cyanovaleric acid) (1.4 mg, 0.0048 mmol) were dissolved in 1.5 mL of *p*-dioxane. This mixture was transferred to a Schlenk flask containing a magnetic stir bar. The oxygen was removed by purging with nitrogen for 15 min. The mixture was heated in an oil bath at 80 °C for 24 h. Then, the polymerization was stopped by cooling the solutions in an ice bath. Removal of unreacted VAc was achieved by dissolving in dichloromethane and precipitating in diethyl ether for three times. Finally, the product was dissolved again in DCM (dichloromethane) and dried under reduced pressure. This purification method led to well purified polymers instead of the use of only reduced pressure after precipitation. Star copolymers were obtained as a white solid (54%). M_n GPC_ = 94,300 g/mol Ð = 1.04, Table 2, sample (PNVCL_99_-*b*-PVAc_21_)_6_.

^1^H NMR (CDCl_3_, TMS, δ, ppm): 5.0–4.8 (bs, –C*H*OCOCH_3_ from PVAc), 4.65–4.12 (bs, –NC*H* from PNVCL), 3.50–2.96 (bs, –NC*H*_2_ from PNVCL), 1.95 (br, 3H, C*H*_3_–CO–), 1.9–1.0 (bm, NCH_2_C*H*_2_C*H*_2_C*H*_2_ of caprolactam ring and C*H*_2_ of backbone from caprolactam and PVAc).

#### 2.3.4. Preparation of Star PNVCL-*co*-PNVP Copolymers with Six Arms

The following procedure is typical for the synthesis of star polymers (PNVCL-*co*-PNVP)_6_: CTA (50 mg, 0.0382 mmol), NVCL (3 g, 21.58 mmol), NVP (0.5 g, 4.5 mmol) and 2,2′-azobis(4-methoxy-2,4-dimethyl valeronitrile, V-70) (3.7 mg, 0.012 mmol) were dissolved in 2.0 mL of *p*-dioxane and mixed in a Schlenk flask containing a magnetic stir bar. The oxygen was removed by purging with nitrogen for 15 min. The mixture was heated in an oil bath at 30 °C for 8 h. Then, the polymerization was stopped by cooling the solutions in an ice bath. Removal of unreacted NVCL and NVP was achieved by dissolving in dichloromethane and precipitating in diethyl ether for three times. Finally, the product was dissolved again in DCM and dried under reduced pressure. The star copolymer product was obtained as a white solid (45%). M_n GPC_ = 28,000 g/mol Ð = 1.01, Table 3, sample (PNVCL_0.83_-*co*-PNVP_0.17_)_6_. 

^1^H NMR (CDCl_3_, TMS, δ, ppm): 4.65–4.10 (bs, –NC*H* from PNVCL), 4.10–3.55 (bs, –NC*H* from PNVP), 3.50–2.75 (bs, NC*H*_2_ from PNVCL and PNVP), 2.6–2.3 (bs, COC*H*_2_, from PNVCL and PNVP), 1.9–1.0 (bm, C*H*_2_ of caprolactam ring and backbone from PNVCL and PNVP).

#### 2.3.5. Preparation of Star [(PNVCL-*co*-PNVP)-*b*-(PVAc))] Block Copolymers with Six Arms

Sample (PNVCL_0.83_-*co*-PNVP_0.17_)_6_ (used as a macroCTA) (0.27 g, 0.0096 mmol), VAc (0.2 g, 2.32 mmol) and 4,4-azobis(4-cyanovaleric acid) (2.6 mg, 0.009 mmol) were dissolved in 1.5 mL of *p*-dioxane. This mixture was transferred to a Schlenk flask containing a magnetic stir bar. The oxygen was removed by purging with nitrogen for 15 min. The mixture was heated in an oil bath at 80 °C for 24 h. Then, the polymerization was stopped by cooling the solutions in an ice bath. Removal of unreacted VAc was achieved by dissolving in dichloromethane and precipitating in diethyl ether for three times. Finally, the product was dissolved again in DCM and dried under reduced pressure. Star copolymers were obtained as a white solid (22%). M_n GPC_ = 37,460 g/mol Ð = 1.1, Table 3, sample ((PNVCL-*co*-PNVP)-*b*-PVAc)_6_-1.

^1^H NMR (CDCl_3_, TMS, δ, ppm): 5.0–4.8 (bs, –C*H*OCOCH_3_ from PVAc), 4.65–4.10 (bs, –NC*H* from PNVCL), 4.10–3.55 (bs, –NC*H* from PNVP), 3.50–2.75 (bs, NC*H*_2_ from PNVCL and PNVP), 2.6–2.2 (bs, COC*H*_2_, from PNVCL and PNVP), 1.95 (br, 3H, C*H*_3_–CO–), 1.9–1.0 (bm, C*H*_2_ of caprolactam ring and backbone from PNVCL and PNVP).

#### 2.3.6. Preparation of Star (PVAc) Polymers with Six Arms

The following procedure was used for the synthesis of star polymer (PNVCL_30_)_6_: CTA-1 (0.12 g, 0.092 mmol), VAc (2 g, 23.2 mmol) and 4,4-azobis(4-cyanovaleric acid) (2.5 mg, 0.009 mmol) were dissolved in 1.5 mL of *p*-dioxane. This mixture was transferred to a Schlenk flask containing a magnetic stir bar. The oxygen was removed purged with nitrogen for 15 min. The mixture was heated in an oil bath at 60 °C for 12 h. Then, the polymerization was stopped by cooling the solutions in an ice bath. Removal of unreacted VAc was achieved by dissolving in dichloromethane and precipitating in diethyl ether for three times. Finally, the product was dissolved in DCM and removed under reduced pressure. This purification yielded polymers with higher purity than were obtained with the use of reduced pressure alone. Star (PVAc)_6_ polymer product was obtained as a white solid (65%). Table S1, Entry 3, (PVAc_30_)_6_, M_n GPC_ = 11,980 g/mol, Ð = 1.2.

^1^H NMR (CDCl_3_, TMS, δ, ppm): 1.73 (br, 2H, –C*H*_2_CH–), 1.95 (br, 3H, –C*H*_3_–CO–), and 4.80 (bs, 1H, –CH_2_–C*H*–).

#### 2.3.7. Preparation of Star (PVAc-*b*-PNVCL) Block Copolymers with Six Arms

The general procedure for the preparation of star block copolymers was as follows: star (PVAc_30_)_6_-macroCTA (0.20 g, 0.17 mmol), NVCL (1.0 g, 7.2 mmol), and 4,4-azobis(4-cyanovaleric acid) (1.5 mg, 0.0054 mmol) were dissolved in 1.0 mL of *p*-dioxane. This mixture was transferred to a Schlenk flask containing a magnetic stir bar. The oxygen was removed via purging with nitrogen for 15 min. The mixture was heated in an oil bath at 65 °C for 48 h. Then, the polymerization was stopped by cooling the solutions in an ice bath. The residual NVCL monomer was removed adding a three-fold excess of diethyl ether. Star (PVAc_22_-*b*-(PNVCL_11_))_6_ polymer product was obtained as a white solid. Table 4, Entry 1, M_n GPC_ = 17,420 g/mol, Ð = 1.2, molar ratio: PVAc:PNVCL 11:89, LCST = 24 °C.

^1^H NMR (CDCl_3_, TMS, δ, ppm): 5.02–4.75 (bs, –C*H*OCOCH_3_ from PVAc), 4.72–4.23 (bs, –NC*H*), 3.4–2.95 (bs, –NC*H*_2_), 2.65–2.26 (bs, –COC*H*_2_), 2.08–1.95 (br, 3H, –C*H*_3_–CO–), 1.95–1.1 (bm, NCH_2_C*H*_2_C*H*_2_C*H*_2_ of caprolactam ring of PNVCL and C*H*_2_ of backbone from PNVCL and PVAc).

#### 2.3.8. Preparation of Star [PVAc-*b*-(PNVCL-*co*-PNVP)] Block Copolymers with Six Arms

The general procedure for the preparation of star diblock copolymers starting with star PVAc-macroCTA was as follows: star PVAc-macroCTA (0.25 g, 0.26 mmol), NVCL (0.7 g, 5.04 mmol), NVP (0.1 g, 0.8 mmol) and 4,4-azobis(4-cyanovaleric acid) (1.5 mg, 0.0054 mmol) were dissolved in 1.5 mL of *p*-dioxane. This mixture was transferred to a Schlenk flask containing a magnetic stir bar. The oxygen was removed via purging with nitrogen for 15 min. The mixture was heated in an oil bath at 65 °C for 48 h. Then, the polymerization was stopped by cooling the solutions in an ice bath. The residual NVCL monomer was removed by adding a three-fold excess of diethyl ether. Star [PVAc_30_-*b*-(PNVCL_28_-*co*-PNVP_17_)]_6_ polymer product was obtained as a white solid (30%). Table 4, Entry 2, M_n GPC_ = 42,290 g/mol, Ð = 1.1.

^1^H NMR (CDCl_3_, TMS, δ, ppm): 5.0–4.8 (bs, –C*H*OCOCH_3_ from PVAc), 4.65–4.12 (bs, –NC*H* from PNVCL), 4.09–3.66 (bs, –NC*H* from PNVP), 3.50–2.96 (bs, –NC*H*_2_ from PNVCL and PNVP).

#### 2.3.9. Removal of Xanthate End Groups from Star (PNVCL)_6_ Polymers

In a Schlenk flask containing a magnetic stir bar, star (PNVCL_52_)_6_ polymers (Table S2, Entry 2, M_n GPC_ = 43,100 g/mol, Ð = 1.03, 0.167 g, 0.0038 mmol) were dissolved in 1.5 mL of *p*-dioxane and ACVA (10 mg, 0.038 mmol) was added. The solution was de-oxygenated by bubbling nitrogen for 20 min at room temperature. Then, the flask was placed in an oil bath preheated at 80 °C for 24 h. The reaction was stopped by cooling to room temperature. The *p*-dioxane was removed under reduced pressure and the product was dissolved in the minimum amount of DCM. The by-products derived from ACVA were removed adding a three-fold excess of diethyl ether and the star polymer was recovered as a precipitate. For the case of low molecular weight (PNVCL)_6_ polymers (M_n_ ˂ 5000 g/mol), an additional purification step was required. After purification with diethyl ether, the ACVA-treated polymer was dissolved in methanol and kept under stirring for 10 min. The precipitate was discarded and the methanol was removed under reduced pressure.

#### 2.3.10. Preparation of Aggregates from Star Block Copolymers

Aggregates of star polymers “micelles” were prepared by dispersing (1.0 mg/mL) of the star block-copolymers in water and stirring at room temperature from 24 to 48 h until an optical clear dispersion was observed.

#### 2.3.11. Preparation of MTX Loaded Aggregates

The incorporation of MTX into star self-assembled (PVAc-*b*-(PNVCL-*co*-PNVP))_6_ or star ((PNVCL-*co*-PNVP)-*b*-(PVAc))_6_ self-assembled aggregates was achieved by the dialysis method. Briefly, the star-shaped block copolymer (30 mg) and MTX (3 mg) were dissolved in DMAC (1 mL). The mixture was stirred at room temperature for 0.5 h and then the solution was added to deionized water drop-wise (5 mL) and stirred for 1 h. The solution was dialyzed against deionized water for 8 h at 25 °C with a dialysis membrane of MWCO (molecular weight cut off) 3.5 kD, the water was replaced every 4 h. Finally, the sample was lyophilized for 24 h.

To determine the loading content, the MTX loaded star polymeric aggregates (2 mg) were dissolved in 10 mL of DMF and were analyzed by UV-vis spectroscopy at a wavelength of 303 nm. The MTX concentration was calculated by comparing the absorbance to a standard calibration curve obtained from MTX in DMF solutions. The drug loading content (LC) was calculated according to the following formula:

LC (wt %) = (weight of loaded MTX/weight of loaded aggregates) × 100%

#### 2.3.12. In Vitro Drug Release

MTX-loaded star block copolymers (5 mg) were suspended in phosphate buffer saline (PBS) solution (3 mL) (0.1 M, pH = 7.4) and stirred for 1 h. Then the dispersion was transferred to a dialysis bag. The dialysis bag was sealed and placed in 100 mL of PBS at 33, 37 or 40 °C. At predetermined time intervals, 3 mL of PBS were withdrawn from the release medium and replaced with fresh PBS. The released amount of MTX was determined by UV spectroscopy by measuring the absorbance at 303 nm. By comparing the amount of the released drug and total drug loading, a cumulative release was obtained. All loading and release experiments containing methotrexate were carried out in the dark.

## 3. Results and Discussion

### 3.1. Polymerization of NVCL Using R-Hexafunctional Xanthate RAFT Agent

Previously, the controlled polymerization of star-shaped PNVCL polymers with four and six arms was reported using a trithiocarbonate multifunctional RAFT agent [33,34]. The polymerization results indicated that no linear relationship between the M_n_ and conversion was obtained, which is typical for a well behaving RAFT polymerization; nevertheless, the living character of the polymers was demonstrated by obtaining block copolymers with ethyl hexyl acrylate [34]. It is well known that trithiocarbonates are not the best RAFT agents for polymerization of less activated monomers such as NVCL and VAc, as opposed to xanthates that are well known RAFT agents for controlled polymerization of VAc [35,37,41]. Therefore, in the present study, the synthesis of star-shaped PNVCL polymers having six arms has been undertaken using an R-hexafunctional xanthate agent.

**Figure 1:**
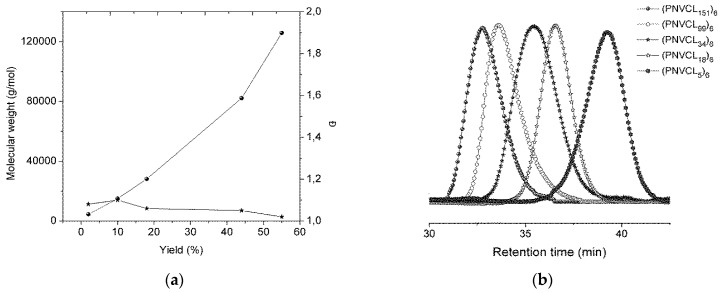
(**a**) Evolution of the molecular weight with conversion for the reversible addition–fragmentation chain transfer (RAFT) solution polymerization of PNVCL (poly(*N*-vinylcaprolactam) at 30 °C in the presence of the hexafunctional CTA-1. [NVCL]_o_ = 14.4 mol/L, [NVCL]_o_:[CTA]_o_ ratio = 2320. (**b**) Normalized GPC (gel permeation chromatography) traces of star PNVCL polymers in THF (tetrahydrofuran).

Initially, the polymerization reaction was carried out using the initiator ACVA at 70 °C, but star polymers were obtained in very low yield. Moreover, GPC traces showed evidence of by-products at higher retention times, which probably come from unreacted hexafunctional CTA. These by-products were also observed in the ^1^H NMR spectrum. Finally, the polymerization reaction was achieved at 30 °C in the presence of the initiator 2,2′-azobis(4-methoxy-2,4-dimethyl valeronitrile) (V-70). It is important to mention that the half life time of ACVA at 70 °C and V-70 at 30 °C is the same [58] Figure 1a shows the evolution of the molecular weight as a function of conversion. It was observed that the M_n_ increased with the polymerization time, and the dispersity behavior is typical of a controlled RAFT polymerization (Ð = M_w_/M_n_ = ≤1.1; M_w_ (weight-averaged molecular weight); M_n_ (number-averaged molecular weight)). In Figure 1b, the overlay of GPC chromatograms shows unimodal peaks with narrow dispersity. The experimental data are presented in Table 1. The M_n_ values obtained by GPC are systematically lower than the theoretical values. An explanation for this behavior could result from the inaccuracy of GPC for the determination of real molecular weights of highly branched molecules since the calibration of molecular weight detector was performed using a linear molecule. Furthermore, the used dn/dc value for calculations evolved from a report on linear PNVCL. Figure S5 shows a typical ^1^H NMR spectrum of star (PNVCL)_6_ polymers.

### 3.2. Solution Properties and Thermosensitivity of Star (PNVCL)_6_ Polymers

**Figure 2:**
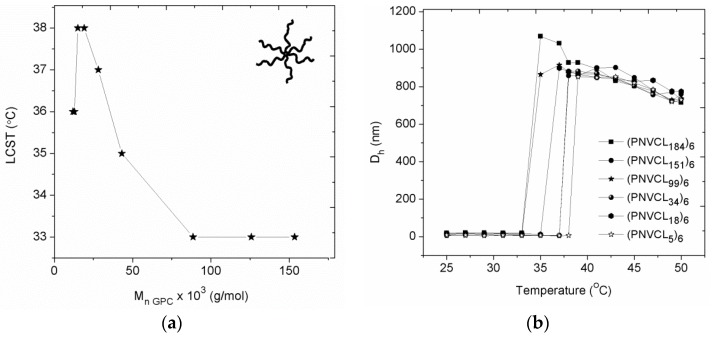
Dynamic light scattering (DLS) measurements (*c* = 1 mg/mL in water): (**a**) effects of molecular weight on the lower critical solution temperature (LCST) of star (PNVCL)_6_ polymers; and (**b**) evolution of the D_h_ of the (PNVCL)_6_ polymers as a function of the temperature.

The behavior of linear PNVCL in water corresponds to polymers of type I with a Flory–Huggins behavior in which the transition temperature value (LCST) of polymers decreases with increasing polymers chain length [1,33]. The thermosensitivity behavior of star-shaped PNVCL polymers having six arms was studied in aqueous solution by monitoring the aggregation behavior by heating using DLS, which was ascribed to the LCST of the system. The values for the LCST of 1 wt % aqueous solutions of the star (PNVCL)_6_ polymers as a function of the molecular weight are shown in Figure 2a. Detailed experimental conditions are provided in the Supplementary Materials (Table S3). The decrease of M_n_ of the star (PNVCL)_6_ polymers from 153,400 to 15,000 g/mol resulted in an increase on LCST from 33 to 38 °C. It can be observed that the samples (PNVCL_184_)_6_, (PNVCL_151_)_6_ and (PNVCL_106_)_6_ exhibited a LCST of 33 °C, which is reasonable since the molecular weight of the polymeric arms range from 26,000 to 15,000 g/mol [34]. The samples (PNVCL_23_)_6_ and (PNVCL_18_)_6_ (3200–2500 g/mol by arm) exhibit a LCST of 38 °C. For samples (PNVCL_15_)_6_ and (PNVCL_14_)_6_) with M_n_ from 12,800 and 11,880 g/mol, respectively, a LCST value of 36 °C was observed. In general terms, as the molecular weight of the star decreases, the transition temperature increases, however, the expected trend is not observed for all samples. Star (PNVCL)_6_ polymers with M_n_ ˂ 6000 g/mol were totally insoluble in water. This fact seems to be related to the relative content of the xanthate group (totally insoluble in water) as compared to those shorter polymer chains. On the other hand, the evolution of the hydrodynamic diameter (D_h_) of the (PNVCL)_6_ star polymers as a function of the temperature is shown in Figure 2b. Above the LCST, the size of the aggregates formed, reached a maximum value (~1060 to 860 nm) and then decreased smoothly while the temperature continues to rise. The resultant aggregates are very similar in size, when it reaches its maximum value, above the LCST. The sample (PNVCL_184_)_6_ reaches a D_h_ = 1060 nm but the rest of the star samples reached a D_h_ = 860 nm. This behavior is different from that previously reported for other PNVCL star polymers [33]. Generally, the D_h_ above the LCST (maximum value) of the aggregates is related to the molecular weight: lower M_n_, yield aggregates with smaller size [33].

To evaluate the influence of the xanthate end group on the LCST, four samples of (PNVCL)_6_ were subjected to removal of this group by using the method of radical-induced reduction [18]. Thus, by adding an excess of the initiator ACVA and heating at 80 °C for 24 h, the removal of the xanthate group was achieved, resulting in the introduction of the initiator fragment (4,4-azobis(4-cyanovaleric acid) at the end of the star polymer arms [25].

By means of ^1^H NMR spectroscopy it is difficult to obtain evidence of the removal of the xanthate group from these star polymers, the peaks at chemical shifts of 4.43 and 4.15 ppm from xanthates moiety are completely overlapping with other peaks of the samples. To ensure the extent of end-group removal, the polymers were dissolved in THF and analyzed by UV-vis spectroscopy. Figure S6a shows the absorption band at 285 nm characteristic of the xanthate group in the (PNVCL)_6_ star polymers. The sample (PNVCL_52_)_6_ (M_n_ = 43,100 g/mol) exhibits a lower absorption value compared with the sample (PNVCL_14_)_6_ (M_n_ = 11,880 g/mol), since the concentration of the thiocarbonyl fragment in the polymer decreases as the molecular weight increases. In comparison, a complete disappearance of the absorption was observed for these polymers after reaction with ACVA (see Figure S6a).

THF GPC chromatograms obtained for (PNVCL)_6_ polymers after reaction with ACVA were unimodal and no other peak was detected. This confirms that removal of the xanthate group was carried out under controlled conditions and no star-star coupling reactions were obtained. With the ACVA treatment a relevant change in the molecular weight of the polymer is not expected, however, it was observed for all samples that the GPC peak after reaction with ACVA shifts slightly to a longer retention time, as a consequence of the decrease in the molecular weight of the polymer. For instance, the M_n GPC_-value of the sample (PNVCL_52_)_6_ changed before and after reaction with ACVA from 43,100 to 39,300 g/mol (Figure S6b). In addition, the M_n GPC_-value of the sample (PNVCL_14_)_6_ changed before and after reaction with ACVA, from 11,880 to 8900 g/mol (see Figure S7 in Supplementary Materials). After the reaction with ACVA, the PNVCL star polymer arms have now the functionality of the initiator (COOH) so the interactions with the column of the GPC shall be stronger, increasing their retention time. Polymers with M_n_ ˂ 15,000 g/mol (~2500 g/mol by arm) showed an increase in their transition temperature after this reaction. As an example, the sample (PNVCL_14_)_6_ showed an LCST of 36 °C before xanthate removal. After reaction with ACVA the LCST of this sample is 39 °C. In other case, the sample (PNVCL_4_)_6_ initially was not soluble in water; after reaction with ACVA the now fully water soluble star polymer showed an LCST of 31 °C. Since the LCST is related with the energy needed to break water-polymer interactions, the introduction of carboxylic acid groups at the chain-ends of the six arms, resulted in additional hydrogen bonding that needs to be broken to induce hydrophobic polymer-polymer interactions that leads to aggregation. Detailed experimental conditions are provided in the Supplementary Materials (Table S2). In the literature, there are reports on the influence of the RAFT agent core as well as the end groups on the LCST of polymer [59,60,61]. Whittaker et al. [62] studied the influence of the hydrophobic end group on the LCST of star poly(*N*-isopropylacrylamide) polymers. They found significant influence from star core and benzyl end groups on the LCST. After hydrolysis, the linear PNIPAM exhibited an LCST of 35 °C, regardless of molecular weight. Probably the presence of hydrophilic and hydrophobic end groups cancels effects on the LCST.

**Figure 3:**
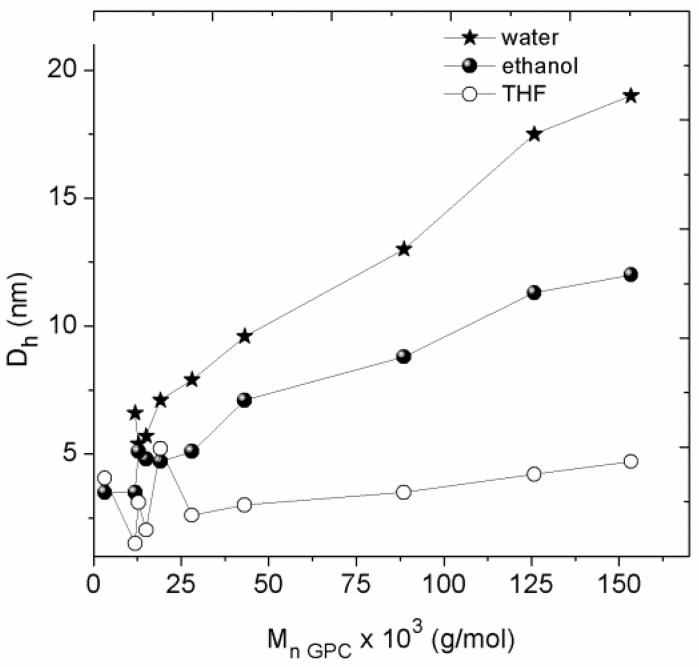
DLS measurements. Evolution of the D_h_ of the star (PNVCL)_6_ polymers as a function of the molecular weight (*c* = 1 mg/mL).

The solution characteristics of the star PNVCL polymers were studied by DLS also in different solvents: water, ethanol and THF at 25 °C (1 mg/mL) (Figure 3 and Table S3 in the Supplementary Materials). In general terms, it was observed that the D_h_ increases with increasing M_n_ of the polymeric star. The lowest D_h_’s were observed for the stars polymers dissolved in THF (1.5 to 4.7 nm). In ethanol, the D_h_ increased in comparison to the D_h_ in THF (4.8 to 12 nm); while in water, the D_h_ of the star polymers ranged from 5 to 19 nm at 25 °C. It is possible that in ethanol, the PNVCL star polymers expand due to the polarity of the solvent and in water a maximum expansion is attained. For instance, the extended chain length of the PNVCL star polymer with M_n_ = 88,580 g/mol, considering that each arm has 106 monomer units and two arms represents an expanded star diameter, results in 212 carbon-carbon units. A simple calculation taking 0.254 nm for C–C, results in 53.8 nm for a totally (non-realistic) extended star polymer. On the other side, the star polymer in a good solvent (ethanol) with partially expanded star arms (R_h_ 4.4 nm), results in an end to end distance (h) of 14 nm. For this calculation h was taken as defined in [63], and R_g_ = R_h_ × 1.2 was used, as the average value for stars with monodisperse arms [64]. The result h = 14 nm match very close the measured D_h_ of 13 nm in water (see calculations of all stars in Table S3). Therefore, the sizes observed in water do not imply aggregation, but rather the maximum expansion of star arms (and the formation of unimers instead aggregates).

### 3.3. Synthesis of Star Poly(N-vinylcaprolactam)-b-poly(vinyl acetate) Block Copolymers

**Scheme 1:**
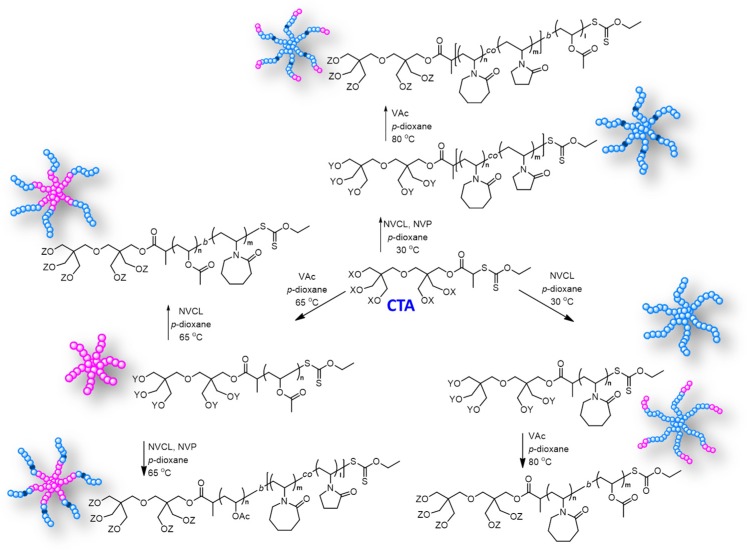
Synthesis of hexafunctional star polymers and block copolymers based on PNVCL using a multifunctional xanthate as a RAFT agent.

**Scheme 2:**
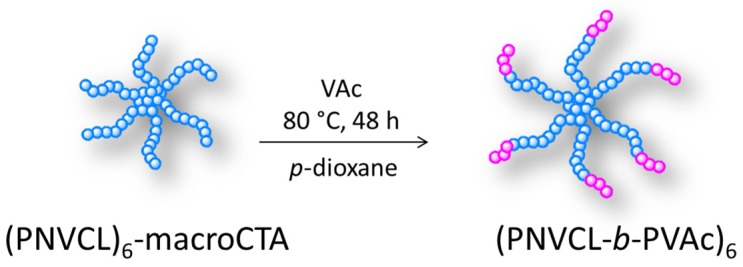
Synthesis of star (PNVCL-*b*-PVAc)_6_ copolymers.

**Figure 4:**
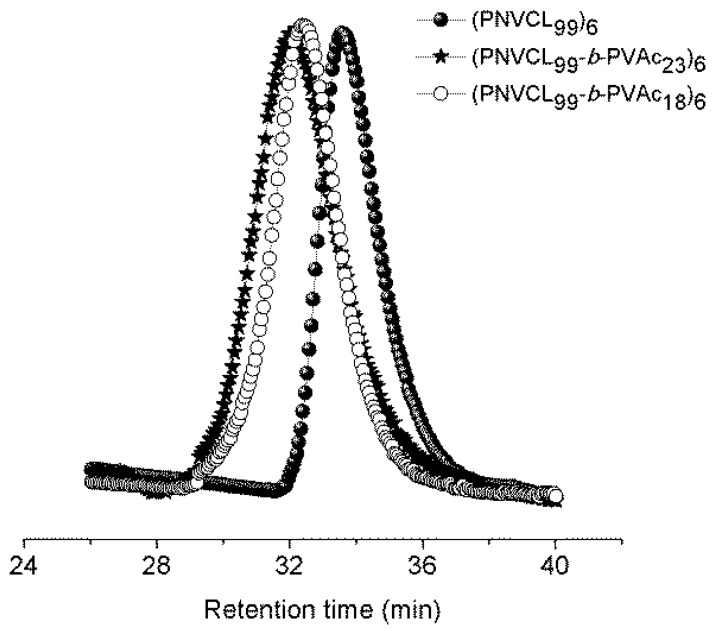
Normalized Gel permeation chromatography (GPC) traces in THF for star (PNVCL)_6_ polymers and star (PNVCL-*b*-PVAc)_6_ block copolymers.

**Figure 5:**
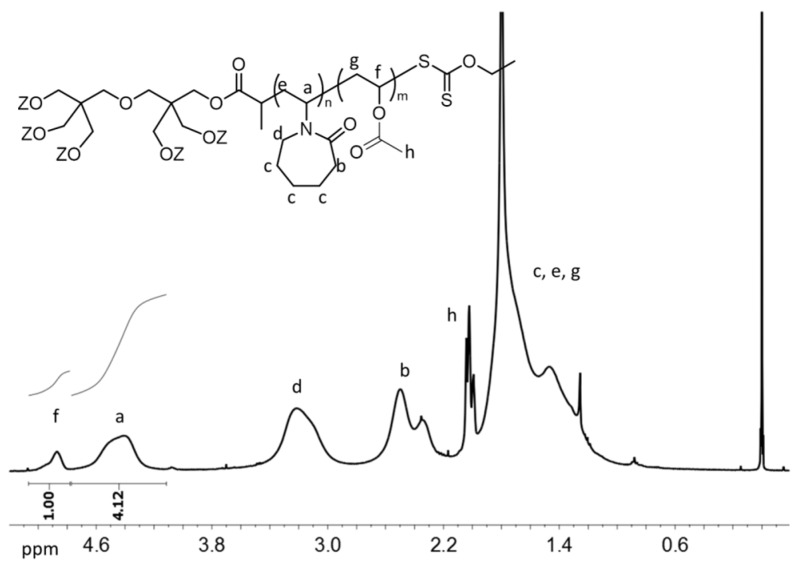
^1^H NMR (400 MHz) spectrum of a star (PNVCL-*b*-PVAc)_6_ copolymer in CDCl_3_ (Table 2, sample (PNVCL_99_-*b*-PVAc_21_)_6_, M_n GPC_ = 94,300 g/mol, Ð = 1.04, 20% of PVAc).

The star (PNVCL)_6_ polymers were used as a macro-CTA’s for the RAFT polymerization of vinyl acetate (VAc) to synthesize six-arm star (PNVCL-*b*-PVAc)_6_ block copolymers (Scheme 1). It is important to note that the PVAc hydrophobic segment in the copolymer is inserted at the end of the polymeric chain (see Scheme 2). Initially, the reaction was explored at 30 °C using V70 as initiator, and at 60 and 70 °C using ACVA as initiator, but no copolymer was obtained. This reaction was only successful at 80 °C using ACVA for 24 h. The initial polymerization conditions chosen were based on the reactivity of PNVCL macro-radicals (reinitiating Z-group) and low reactivity of VAc monomer [25,38]. When star PNVCL with initial M_n_ = 82,600 g/mol was used as macro-CTA (sample (PNVCL_99_)_6_), under favorable polymerization conditions (80 °C), diblock copolymers with increased M_n_ of 94,500, 94,300 and 93,700 g/mol were obtained. The molar content of PVAc in these copolymers was 24, 20 and 13 mol % respectively. An evident shift can be seen in GPC chromatograms for these copolymers when compared with the chromatograms of the precursor polymers, indicating that the initiation efficiency of the star (PNVCL)_6_-macroCTA was high (see Figure 4). Likewise, when star PNVCL with M_n_ = 19,100 g/mol was used as macro-CTA (sample (PNVCL_23_)_6_), diblock copolymers with increased M_n_ of 20,200 g/mol and 24,700 g/mol were obtained. The GPC peaks for these copolymers are shown in Figure S8 in the Supplementary Materials. The ^1^H NMR spectrum (see Figure 5) of a star (PNVCL-*b*-PVAc)_6_ copolymer reveals two of the more important signals of the PVAc: the peak at a chemical shift of 1.95 ppm “h” corresponds to the methyl group next to the carbonyl and the peak at 4.80 ppm “f” corresponds to the methine of PVAc. The molar composition of each segment of the star block copolymers was determined using ^1^H NMR spectroscopy by comparing the integration values of the methine peak of the PVAc block at 4.8 ppm “f” and the methine peak of the PNVCL block at 4.44 ppm “a”. Figure 5 shows the ^1^H NMR spectrum of a star block copolymer with 20 mol % of PVAc.

The preparation of star-like architectures in which the hydrophobic segment is located at the end of the polymeric chain is rare in the literature [33,34,66,67]. It can be expected that in aqueous solution these kinds of polymers would tend to form flower-like micellar aggregates of the star block copolymers, or large associations between these segments leading to the formation of physical networks [66,67]. In a previous work, star PNVCL block copolymers prepared with the hydrophobic poly(ethyl hexyl acrylate) (PHEA) located at the end of the polymer chains assembled to form larger aggregates in aqueous solution (even with very low content of PHEA), probably due to the adhesive properties of PEHA.

However, there is another possibility that consists in the formation of unimolecular micelles. Lin et al. [68] reported the formation of unimolecular micelles from star-like poly(acrylic acid)-*b*-polystyrene starting from a β-cyclodextrin core. The hydrophobic polystyrene segment was located in the shell of the star architecture. The size of these aggregates was obtained from TEM and DLS (~15 nm).

**Figure 6:**
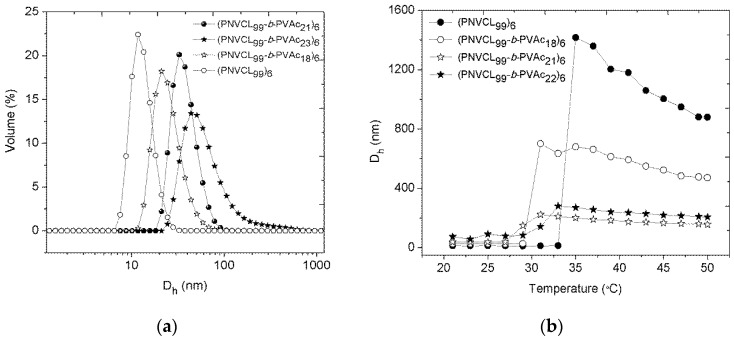
DLS measurements (*c* = 1 mg/mL in water) for star (PNVCL-*b*-PVAc)_6_ block copolymers: (**a**) D_h_ of (PNVCL_99_)_6_ and the corresponding (PNVCL_99_-*b*-PVAc)_6_ block copolymers, measured at 20 °C; and (**b**) evolution of the D_h_ of the (PNVCL_99_)_6_ and (PNVCL_99_-*b*-PVAc)_6_ as a function of the temperature.

To identify by DLS which type of aggregates were formed, the D_h_ in aqueous dispersion of star (PNVCL)_6_ and star (PNVCL-*b*-PVAc)_6_ were compared, as measured at 20 °C (*c* = 1 mg/mL), a temperature below the LCST of the star polymers (see Figure 6a,b). 

**Figure 7:**
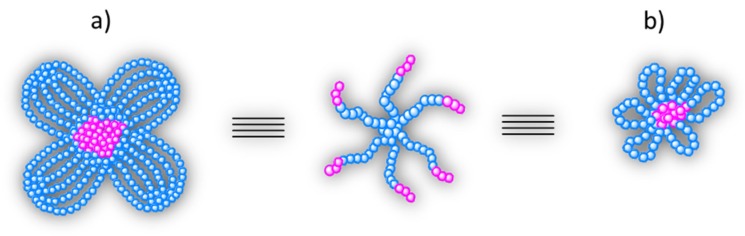
Self-assembly in aqueous solution of star (PNVCL-*b*-PVAc)_6_ block copolymers: (**a**) multiple star micelles; and (**b**) single flower-like micelle.

By DLS, it was observed that the D_h_ of the star copolymers increased slightly with the incorporation of a small amount of hydrophobic PVAc segment compared with their parent star (PNVCL)_6_ polymers. Data are presented in Table 2. As an example, the D_h_ of the sample (PNVCL_23_)_6_ (Table 2) was ~7 nm, and the D_h_ of the block copolymers with 8% of PVAc is ~8 nm; in addition, the D_h_ of the sample (PNVCL_99_)_6_ (Table 2) was ~13 nm, and the D_h_ of the block copolymers with 20 and 13% of PVAc was ~38 and 25 nm (see Figure 6a); while for the sample (PNVCL_51_-*b*-PVAc_2_)_6_, the D_h_ is practically unchanged for the homopolymer and copolymer containing 5% of PVAc (change from 9 to 9.8 nm). An increase in the content on PVAc in the range between 20% and 26% resulted in values of D_h_ doubling the D_h_ of the starting PNVCL stars without PVAc (see Table 2). When the D_h_ for the block copolymer doubles the D_h_ of the starting PNVCL star without PVAc, the aggregate proposed in Figure 7a (multiple star micelles) is totally appropriate; whereas Figure 7b proposes an aggregate (flower-like micelle) when the D_h_ of the block copolymer practically remains unchanged when compared to the D_h_ of the starting PNVCL star without PVAc. Figure S9 shows the ^1^H NMR spectrum of star ((PNVCL_99_-b-PVAc_21_)_6_) copolymer aggregates in deuterium oxide (D_2_O). Only the peaks attributed to the PNVCL units are observed in the spectrum (identified as “a, d, b, c and e”). The peaks attributed to the PVAc (at 1.95 and 1.73 ppm from to the methyl and methylene hydrogens) disappear from the spectrum. This is because the hydrophobic PVAc segment is concentrated in the core of the flower-like micelle; consequently, its molecular mobility is reduced.

By comparing these results with the self-assembly reported for linear PNVCL-*b*-PVAc block copolymers, it was found that linear block copolymers form spherical micelles (*c* = 1 mg/mL) [51]. Furthermore, by decreasing the content of PVAc in the block copolymer, a noticeable increase in the size of micelles was observed (copolymers with 30 and 55 mol % of PVAc form micelles of 123 and 59 nm respectively). The formation of single flowerlike micelles is not reported for linear PNVCL-*b*-PVAc block copolymers; however, it is observed in the case of other amphiphilic star polymers, although in the literature there are a few examples [66,67,68,69,70].

On the other hand, the effect of the PVAc hydrophobic segment in the transition temperature is evident, even when the content on PVAc is low. The LCST for star (PNVCL_23_)_6_ polymer was 38 °C and shifted to 30 and 24 °C for copolymers with 8 and 26 mol % of PVAc (samples (PNVCL_23_-*b*-PVAc_2_)_6_ and (PNVCL_23_-*b*-PVAc_11_)_6_, Table 2). Likewise, the LCST for star (PNVCL_99_)_6_ polymers were 33 °C and shifted to 29 °C for copolymers with 13 mol % of PVAc. That means, the transition temperature in the copolymer depends on both: the PVAc content and the M_n_ of the precursor homopolymeric star PNVCL. Detrembleur et al. [51,52] prepared linear PVAc-*b*-PNVCL copolymers with PVAc content of 30 to 55 mol %. The transition temperatures reported were from 26.5 to 29.5 °C depending on their PVAc content.

Another issue is that it was observed that the D_h_ of the star (PNVCL-*b*-PVAc)_6_ aggregates formed above their LCST is much lower than its precursor homopolymer (Figure 6b). The D_h_ above the LCST of the precursor star (PNVCL_23_)_6_ and the produced star (PNVCL_23_-*b*-PVAc_2_)_6_ were 853 and ~300 nm, respectively. Likewise, the stability of aggregates of amphiphilic copolymers in aqueous dispersion is fundamental for its application in drug release. With the exception of sample (PNVCL_23_-*b*-PVAc_11_)_6_ (LCST = 24 °C), the rest of the copolymer sample solutions show high stability after four weeks (without agitation) when maintained at room temperature (26 °C, below their LCST) since no signs of precipitation were observed and the solutions were transparent. The stability in aqueous solution of these aggregates was tested by DLS studies. For the sample (PNVCL_99_-*b*-PVAc_21_)_6_, it can be observed that the D_h_ practically does not change in the first four weeks (without agitation) (see Figure S10a in the Supplementary Materials). The aqueous solutions of these copolymers remain transparent (below the LCST) even with a PVAc content of 20 mol %. For the sample (PNVCL_51_-*b*-PVAc_2_)_6_, it can be observed that the D_h_ decreased after four weeks without agitation when maintained at a temperature below the LCST (see Figure S10b in the Supplementary Materials). The great stability of these aggregates is only possible if the star architecture forms both multiple star micelles and single flowerlike micelles as proposed in this study. Accordingly, the PVAc end segments in the star, are connected to the same aggregate, comprised or confined in a core and stabilized by means of hydrophilic PNVCL arms forming a shell and favored by the sticker properties of PVAc.

### 3.4. Synthesis of Star {[Poly(N-vinylcaprolactam)-co-poly(N-vinylpirrolydone]-b-poly(vinyl acetate)} Copolymers

As discussed above, the introduction of the PVAc segment into the copolymer causes a decrease in the transition temperature of the PNVCL. A strategy for having star copolymers with hydrophobic units and higher transition temperatures consists of starting their synthesis from a macro-CTA which in addition to PNVCL contains PNVP. This is because it is reported that the introduction of NVP units into polymers of PNVCL increases the transition temperature as a function of NVP content [71]. The synthesis of the star (PNVCL-*co*-PNVP)_6_ used as a macro-CTA was achieved under the same reaction conditions at which the star (PNVCL)_6_ polymers were obtained. The ^1^H NMR spectrum (see Figure S11, Supplementary Materials) of the star (PNVCL-*co*-PNVP)_6_ copolymer reveals the peak between 4.0 and 3.5 ppm attributed to the methine hydrogen “g” (–CH_2_–C*H*–) from PNVP. The rest of the hydrogens of the PNVP are practically overlapped with the hydrogens of the PNVCL. The molar composition of PNVP in the copolymer was determined using ^1^H NMR spectroscopy by comparing the integration value of the methine peak of the PNVCL at 4.44 ppm and the peak in the range between 3.50 and 2.75 ppm (“c, h”). This peak corresponds to the methylene attached to N in PNVP and PNVCL. After subtracting the integration value for the two hydrogens from PNVCL, from the total value, the molar ratio of PNVP was determined. Figure S11 shows the ^1^H NMR spectrum of the star (PNVCL-*co*-PNVP)_6_ copolymer with 17 mol % of PNVP.

The ^1^H NMR spectrum (Supplementary Materials, Figure S12) of the star [(PNVCL-*co*-PNVP)-*b*-PVAc]_6_ copolymer reveals at 1.95 ppm the “m” peak corresponding to methyl group of PVAc. The molar composition of each segment of the star copolymer was determined using ^1^H NMR spectroscopy by comparing the integration value of the methine peak of the PVAc block at 4.8 ppm, the methine peak of the PNVCL block at 4.44 ppm “b” and the peak at 3.50 to 2.75 ppm “c, h”. In this last peak, the integration value for the methylene of PNVP was obtained after subtracting the integration value for the two hydrogens from PNVCL to the total value. This calculation demonstrated that the star [(PNVCL-*co*-PNVP)-*b*-PVAc]_6_ copolymer contains 48 mol % of PVAc.

Using (PNVCL-*co*-PNVP)_6_ as macro-CTA for the copolymerization with VAc, [(PNVCL-*co*-PNVP)-*b*-PVAc]_6_ copolymers with 10 to 48 mol % of PVAc were prepared. The aqueous solution of the precursor (PNVCL-*co*-PNVP)_6_ showed a LCST of 41 °C. The LCST values for the copolymers decreased to 34 and 27 °C, for star polymers containing 10 and 13.5 mol % of PVAc, respectively. The sample [(PNVCL-*co*-PNVP)-*b*-PVAc]_6_ with 48 mol % of PVAc was not suitable for the preparation of micelles because it formed much larger aggregates in water, nevertheless it is important to note that the GPC traces indicate that the copolymerization reaction is still well controlled when a large amount of VAc is copolymerized (see Figure S13 in the Supplementary Materials). The characteristics of these synthesized star block copolymers are summarized in Table 3.

### 3.5. Synthesis of Star Poly(vinyl acetate)-b-poly(N-vinylcaprolactam) Block Copolymers

Poly(vinyl acetate)-*b*-poly(*N*-vinylcaprolactam) (PVAc-*b*-PNVCL)_6_ block copolymers containing the PVAc in the core of the star were prepared using RAFT polymerization (see Scheme 1). Firstly, the synthesis (PVAc)_6_ of star polymers was carried out using the hexafunctional xanthate CTA-1 described above. The polymerization reaction of VAc was performed in solution at 60 °C in *p*-dioxane using ACVA as initiator. Figure S14 (Supplementary Materials) shows the ^1^H NMR spectrum of star (PVAc)_6_ polymers. The peak at 4.9 ppm was assigned to the methine hydrogen “b” (–CH_2_–C*H*–) from the main chain. The peaks at 1.95 ppm were assigned to the methyl hydrogens “d” (C*H*_3_–CO–), and the peaks at 1.73 ppm were assigned to the methylene hydrogens “c” (–C*H*_2_–CH–). The peaks observed at 4.58 and 1.36 ppm (“a” and “e”) integrate for two and three hydrogens, respectively (come from the hexafunctional CTA) and were used to determine the M_n NMR_ of the PVAc by comparing their integration ratio with the integration of the methine hydrogen “b” (–CH_2_–C*H*–) from PVAc. The star (PVAc)_6_ polymers were obtained with good controls of M_n_ and dispersity and the GPC traces of the polymers showed unimodal distributions (Figure S15 in the Supplementary Materials). The molecular weights and dispersity values for star (PVAc)_6_ polymers are summarized in Table S1 (Supplementary Materials). As an example, the molecular weights from the ^1^H NMR and GPC analyses for the sample (PVAc_17_)_6_ (Table S1, Entry 1) were 8750 and 6600 g/mol, respectively. As can been observed, the molecular weight calculated from ^1^H NMR was higher than that obtained by GPC.

**Scheme 3:**
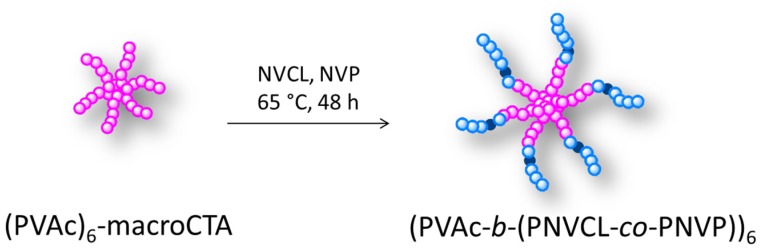
Synthesis of the star [PVAc-*b*-(PNVCL-*co*-PNVP)]_6_ block copolymers.

Subsequently, (PVAc)_6_ polymers (referred to as the (PVAc)_6_-macroCTA) were used to initiate the polymerization of NVCL. The copolymerization reaction with NVCL was achieved at 65 °C in presence of the initiator ACVA. The first PVAc-*b*-PNVCL block copolymer prepared showed a very low transition temperature even though the molar ratio of PVAc remains low as detailed below (Table 4). Therefore, the addition of NVP together with NVCL turned to be necessary to shift the LCST to higher value (see Scheme 3).

**Figure 8:**
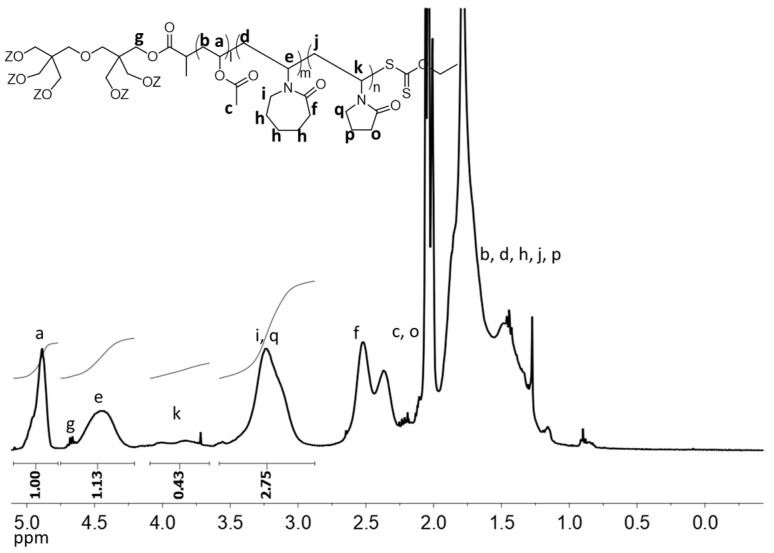
^1^H NMR spectrum (400 MHz) in CDCl_3_ of star-shaped (PVAc-*b*-(PNVCL-*co*-PNVP))_6_ block copolymers. Sample (PVAc_30_-*b*-(PNVCL_28_-*co*-PNVP_17_))_6_, M_n GPC_ = 42,290 g/mol, Ð = 1.1 (Table 4, Entry 2).

A typical ^1^H NMR spectrum in CDCl_3_ of the star (PVAc-*b*-(PNVCL-*co*-PNVP))_6_ copolymers is shown in Figure 8. The peak at 4.45 ppm is attributed to the methine hydrogen “e” (–CH_2_–C*H*–) from PNVCL; the peak (“i, q”) between 3.50 and 2.75 ppm is attributed to the methylene hydrogens (–C*H*_2_–N) from PNVCL and PNVP.

Figure S16 shows the ^1^H NMR of star (PVAc-*b*-(PNVCL-*co*-PNVP))_6_ copolymer aggregates in deuterium oxide (D_2_O). The peaks at 4.45 and 3.75 ppm attributed to the methine hydrogen “a” and “f” (–CH_2_–C*H*–) from PNVCL and PNVP respectively are clearly identified. However, the peak at 1.95 ppm attributed to the methyl hydrogens “c” (C*H*_3_–CO–) from PVAc disappears completely from the spectrum. This is because the hydrophobic segment of PVAc is concentrated in the center of the aggregate and only the peaks of the hydrophilic (PNVP and PNVCL) segments are observed.

It has been reported that chain extension reactions starting from PVAc as macro-CTA is often impractical due to the high reactivity of the growing polymer radicals related to the lack of stabilization of the PVAc macroradical [38]. However, the star copolymer synthesized from the star (PVAc)_6_ polymer as macro-CTA in this study, resulted to be well controlled. The GPC traces for these stars block copolymers showed monomodal peaks (Figure S15a, b in Supplementary Materials). The characteristics of (PVAc-*b*-(PNVCL-*co*-PNVP))_6_ block copolymers synthesized are summarized in Table 4.

Although the transition temperature of the PNVCL can be modulated through of the molecular weight (Flory–Huggins type I polymer), the presence of the hydrophobic block (required for drug encapsulation) decreases the LCST in the amphiphilic copolymer. In addition, large micelles can be formed. PNVP is more hydrophilic than PNVCL. The copolymerization of NVP and NVCL allows to adjust the LCST and in parallel, the sizes of the aggregates decreases, since the balance of hydrophobicity/hydrophilicity is altered. However, the molar ratio of PNVP should be relatively low in the copolymer to have a LCST close to body temperature [35].

As expected, the LCST values for the star block copolymers containing PNVP increased from 24 °C for the star block copolymer without NVP up to 40 °C for the star block copolymer having 11.6 mol % of PNVP.

**Figure 9:**
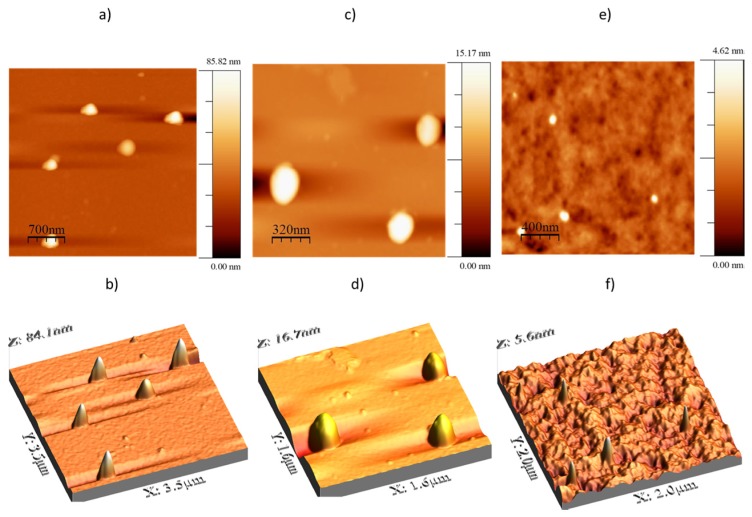
“Height” image and “3D reconstruction“ from AFM of almost spherical aggregates obtained from six-arm star block copolymers on a mica substrate (*c* = 0.02 mg/mL): (**a**,**b**) Sample (PVAc_22_-*b*-PNVCL_11_)_6_, Entry 1, Table 4; (**c**,**d**) Sample [PVAc_30_-*b*-(PNVCL_28_-*co*-PNVP_17_)]_6_, Entry 2, Table 4; and (**e**,**f**) Sample [PVAc_17_-*b*-(PNVCL_10_-*co*-PNVP_7_)]_6_, Entry 3, Table 4.

The aggregation behavior of the star block copolymers having PVAc units in the core of the star changed dramatically when compared to the star block copolymers having PVAc at the end of the arms of the star block copolymers. The star block copolymers having PVAc at the core of the stars showed aggregate sizes in water between 115 and 240 nm (Table 4). The diameter and morphology of these aggregates were confirmed by AFM measurements (Figure 9). The AFM image in Figure 9a,b show aggregates with diameter of ~388 nm corresponding to the sample (PVAc_22_-*b*-PNVCL_11_)_6_ (Table 4, Entry 1). The content of PVAc is 11 mol %, but PNVP is absent in this copolymer. The diameter of the sample [PVAc_30_-*b*-(PNVCL_28_-*co*-PNVP_17_)]_6_ (Table 4, Entry 2) by AFM was 245 nm (Figure 9c,d) and the aggregates are found to be almost spherical. For the sample [PVAc_17_-*b*-(PNVCL_10_-*co*-PNVP_7_)]_6_ (Table 4, Entry 3) the diameter observed was 99 nm (Figure 9e,f). The content of PVAc in the copolymer is 47 mol %, but these star block copolymers contains seven units of PNVP per star arm which contributes to a better dispersability in water, showing smaller aggregates than the star block copolymer without PNVP. In any case, the sizes observed for all three star block copolymers cannot arise from conventional core-shell micelles. Taking as example the star block copolymer that showed the smaller aggregates (D_h_ = 115 nm) and its composition, showing a total of 34 monomer units per arm of the star and calculating the fully extended star (considering two times the arm), it would be 0.254 nm × 34 × 2 = 17.27 nm which is 6.7 times smaller than the observed aggregate size in water. In THF those star block copolymers are fully dispersed, while the star block copolymer without PNVP show in ethanol also strong aggregation (see Table S4 in the Supplementary Materials).

### 3.6. Drug Loading and Thermosensitive MTX Release Using [PVAc-b-(PNVCL-co-PNVP)]_6_ and (PNVCL-b-PVAc)_6_ Copolymers

We recently reported the preparation of star-shaped poly(*N*-vinylcaprolactam)-*block*-poly(ethylhexylacrylate)-*block*-polyethylene glycol (PNVCL-*b*-PEHA-*b*-PEG) aggregates and its application as nanocarriers of MTX [34]. At 33 °C, the release of MTX increases (52% at 24 h) but at 37 °C (above the LCST) the release decreases (30%).

Temperature-sensitive polymeric aggregates prepared from star [poly(vinyl acetate)-*b*-(*N*-vinylcaprolactam-*co*-*N*-vinylpyrrolidone)]_6_ [PVAc-*b*-(PNVCL-*co*-PNVP)]_6_ and [poly(*N*-vinylcaprolactam)-*b*-poly(vinyl acetate)]_6_ (PNVCL-*b*-PVAc)_6_ were tested as delivery systems of the anticancer drug methotrexate (MTX). The MTX-containing polymeric aggregates were generated by using a dialysis method. The amount of encapsulated MTX in the polymeric aggregates was determined from the absorbance of MTX at 308 nm. The release studies were carried out in PBS medium (pH 7.4) at 33, 37 and 40 °C. First, the experimental results with [PVAc-*b*-(PNVCL-*co*-PNVP)]_6_ copolymers will be discussed. These copolymers contain PVAc in the core of the star. In Table 4 (Entry 1), it was observed that block copolymers with only PNVCL and PVAc showed a transition temperature at low values (lower than for pure star (PNVCL)_6_). Likewise, the incorporation of PNVP was absolutely necessary in the copolymer to adjust the transition temperature to physiological conditions for drug delivery purposes. Table 5 summarizes the drug delivery behavior of the copolymers. The content of MTX in the star copolymers ranged from 4.3 to 7 wt %, while the loading efficiency of MTX in the aggregates was higher than 40%.

**Figure 10:**
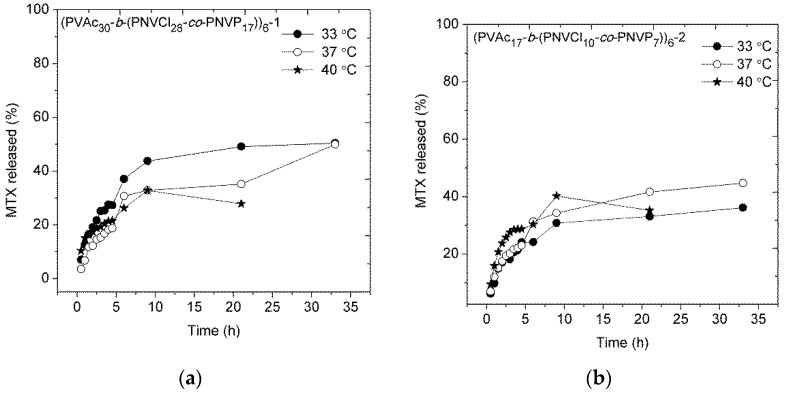
Drug release from micellar aggregates containing MTX: (**a**) Sample (PVAc_30_-*b*-(PNVCL_28_-*co*-PNVP_17_))_6_; and (**b**) Sample (PVAc_17_-*b*-(PNVCL_10_-*co*-PNVP_7_))_6_.

As shown in Figure 10a, sample [PVAc_30_-*b*-(PNVCL_28_-*co*-PNVP_17_)]_6_ (42:47.5:10.5 molar ratio of PVAc:PNVCL:PNVP) releases about 13% of the drug in the first hour, and 49% in 24 h at 33 °C. For this same sample, ~7% of MTX was released in the first hour and 35% in 24 h at 37 °C which means that there are still 51 to 55% of drug entrapped in the polymeric aggregate (Table 5). It can be observed that the rate of release of the drug is higher at 33 °C than at 37 °C. At 40 °C, 32% of the drug is released in 24 h. It is necessary to mention that at 37 and 40 °C, the release of MTX starts with the aggregate in its collapsed form (the sample has a LCST of 36 °C). Most of the drug is entrapped in the core of hydrophobic PVAc of the star covered with collapsed poly(NVCL-*co*-NVP) chains.

For the sample [PVAc_17_-*b*-(PNVCL_10_-*co*-PNVP_7_)]_6_ (47:41.4:11.6 molar ratio of PVAc:PNVCL:PNVP) 33% and 41% of MTX was released, respectively, at 33 and 37 °C at 24 h (Figure 10b). This sample has a LCST of 40 °C. That is, the release takes place below the LCST. In addition, when the release is carried out at 40 °C, 40% of the MTX is released in 24 h. As described above, the stability of aggregates is fundamental for drug delivery applications. According to the literature, the structure, molecular weight of the copolymer, hydrophilic block longer than the hydrophobic block, are key aspects to keep the micelles in a dispersed state [28]. The presence of PNVP in the copolymer contributes to the stability of the aggregates due to it is hydrophilic character during the release process. Above the LCST, no precipitation of micelles was observed in the membrane in the first four hours.

The star (PNVCL-*b*-PVAc)_6_ block copolymers showed a different behavior when they were evaluated as delivery systems of MTX. These copolymers contain PVAc at the end of the arms of the star polymers. In Table S5 (supplementary Materials) are described the properties of MTX-loaded micelles. In aqueous solution, these copolymers are completely transparent (1 mg/mL) and form unimolecular micelles in sizes between 9 and 13 nm (Table 2). The content of MTX in the star copolymers was 4–5 wt % and the loading efficiency of MTX in the aggregates was higher than 35%. The release of MTX was carried at 33 °C, above the transition temperature of the copolymers. For all cases, the drug delivery at 1 and 24 h is faster with respect to the star copolymers with the PVAc block in the core.

As shown in Figure S17, sample (PNVCL_99_-*b*-PVAc_21_)_6_ releases about 24% of the drug in the first hour, and 83% in 24 h at 33 °C; sample (PNVCL_99_-*b*-PVAc_18_)_6_ releases 33% and 71% of MTX at 1 and 24 h, respectively, while sample (PNVCL_51_-*b*-PVAc_2_)_6_ releases about 15% of the drug in the first hour, and 54% in 24 h at 33 °C. It can be deduced that the MTX is trapped less efficiently in the unimolecular micelles since the hydrophobic PVAc segments shields less efficiently its small hydrophobic core. To the contrary, the star [PVAc-*b*-(PNVCL-*co*-PNVP)]_6_ copolymers form aggregates that show a more controlled MTX release behavior.

## 4. Conclusions

The use of a hexafunctional xanthate as CTA allowed for the preparation of a series of star polymers with six arms by RAFT polymerization. Adjusting the polymerization conditions, it was shown that star polymers with homopolymeric PNVCL and homopolymeric PVAc arms could be prepared. Furthermore, NVP could be copolymerized in a controlled way with NVCL to yield star polymers with copolymeric arms. In all cases, the prepared star poly and copolymers show very low dispersity values and predictable molecular weights. The prepared star polymers showed livingness, since they were reactivated for further preparation of star block copolymers. By using this synthetic strategy, a mini library of star polymers was obtained, with hydrophobic PVAc units linked to the core of the star or at the end of the star arms, while the hydrophilic PNVCL arms as well as the copolymeric (hydrophilic) poly(NVCL-*co*-NVP) units were placed in the core as well as at the end of the star polymer arms. In addition, the arm molecular weight of each polymer unit was also changed. The thermoresponsive and aggregation behavior of these star polymers was studied. For instance, star PNVCL homopolymers with molecular weight ˂ 6000 g/mol were insoluble in water; however, after removal of the xanthate groups, by reaction with an excess of ACVA, totally water soluble PNVCL star polymers were obtained. Likewise, PNVCL star polymers with molecular weights between 43,000 and 15,000 g/mol showed a molecular weight dependent LCST that increased after removal of xanthate group. Star PNVCL-*b*-PVAc diblock copolymers with PVAc contents of 5–26% were prepared using the star PNVCL polymers as the macro-CTA. The hydrophobic segment (PVAc) is located at the end of the star arms. Interestingly, the D_h_ value of the aggregates formed in water was almost the same than the size of the corresponding PNVCL star homopolymer when the PVAc content was 5–7%. The star block copolymer may self-assemble into unimolecular micelles (also called single flowerlike micelles). Nevertheless, when the content on PVAc was between 13% and 26%, the D_h_ was approximately double that of the corresponding star PNVCL homopolymers, forming small composite micelles. The LCST of these star block copolymers was much lower (by ~8 °C) as compared with the precursor star PNVCL homopolymers. The decrease in LCST could be counterbalanced by using star PNVCL-*co*-PNVP copolymers as macro-CTA for the preparation of star (PNVCL-*co*-PNVP)-*b*-PVAc block copolymers, showing LCST values between 27 and 34 °C. It was also observed that the D_h_ of these star diblock copolymers is practically unchanged compared with the precursor star PNVCL-*co*-PNVP copolymers. Star PVAc-*b*-PNVCL and PVAc-*b*-(PNVCL-*co*-PNVP) block copolymers were also well prepared. In this case, the hydrophobic segment was located in the core of the star polymers. It was observed that the aggregation behavior of these star block copolymers is totally different with respect to the above mentioned. Without PNVP in the star copolymer arms, big aggregates in water were observed, even for low content of PVAc in the star polymers. With PNVP in the star copolymer it is possible to modulate both the LCST and the D_h_. PVAc-*b*-(PNVCL-*co*-PNVP) and PNVCL-*b*-PVAc block copolymers were used to encapsulate MTX. PVAc-*b*-(PNVCL-*co*-PNVP) copolymers form aggregates that showed a temperature controlled MTX release behavior.

## Figures and Tables

**Table 1:** Results for RAFT polymerization of NVCL in THF at 30 °C in the presence of hexafunctional CTA-1 xanthate-type; PNVCL (poly(*N*-vinylcaprolactam); NVCL (*N*-vinylcaprolactam); CTA-1 (chain transfer agent-1); [NVCL]o:[CTA]o (molar ratio of NVCL to CTA); Conv. (conversion); M_n theo_ (theoretical molecular weight); M_n GPC_ (number-averaged molecular weight determined by gel permeation chromatography); Ð (dispersity =M_w_/M_n_).

Entry	Sample ^a^	[NVCL]_o_:[CTA]_o_	Time (h)	Conv. ^b^ (%)	M_n theo_^c^ (g/mol)	M_n GPC_^d^ (g/mol)	Ð ^d^
1	(PNVCL_151_)_6_	2320	10	55	178,684	125,800	1.02
2	(PNVCL_99_)_6_	2320	6	44	143,200	82,200	1.05
3	(PNVCL_34_)_6_	2320	3	18	59,366	28,100	1.06
4	(PNVCL_18_)_6_	2320	2	10	33,558	15,000	1.10
5	(PNVCL_5_)_6_	2320	1	2	7780	4500	1.08

^a^ The subscript numbers represent the repeating units of PNVCL estimated by using GPC. ^b^ Determined gravimetrically. ^c^ Calculated using equation: M_ntheo_ = M_CTA_ + conv. (M_NVCL_[NVCL]_o_/[CTA]_o_). ^d^ Determined by GPC in THF at 35 °C with RI and LS detectors using polystyrene linear standards for calibration of LS detector. PNVCL dn/dc = 0.109 mL/g [35].

**Table 2:** Characteristics of the star (PNVCL-*b*-PVAc)_6_ copolymers.

Sample ^a^	Precursor Homopolymer				Corresponding Block Copolymer				
	Star (PNVCL)_6_				Star (PNVCL-*b*-PVAc)_6_				
	M_n GPC_ (g/mol) ^b^	Ð ^b^	LCST (°C) ^c^	D_h_^d^ (nm)	M_n GPC_ (g/mol) ^e^	Ð ^e^	PVAc (mol %) ^f^	D_h_^d^ (nm)	LCST (°C) ^c^
(PNVCL_23_-*b*-PVAc_2_)_6_	19,100	1.08	38	7.1 (1.1)	20,200	1.07	8	7.8 (1.3)	30
(PNVCL_23_-*b*-PVAc_11_)_6_					24,700	1.05	26	16.9 (3.5)	24
(PNVCL_51_-*b*-PVAc_2_)_6_	42,200	1.02	35	9 (2.3)	43,100	1.03	5	9.8 (2.6)	28
(PNVCL_99_-*b*-PVAc_21_)_6_	82,600	1.01	33	13.3 (3.6)	94,300	1.04	20	38 (12)	28
(PNVCL_99_-*b*-PVAc_18_)_6_					93,700	1.05	13	25 (11.6)	29

^a^ The subscript numbers represent the repeating units of each PNVCL and PVAc block estimated by using GPC. ^b^ By GPC in THF at 35 °C with RI and LS detector using polystyrene linear standards for calibration of LS detector. ^c^ Determined by DLS (1 mg/mL in water). ^d^ Determined by DLS (1 mg/mL) in water at room temperature. ^e^ By GPC in THF at 35 °C with RI and LS detector using polystyrene linear standards for calibration of LS detector. The dn/dc value used was calculated by using the following formula according to the composition of the copolymer by ^1^H NMR: dn/dc = 0.109(X) + 0.054(Y), where X and Y are the respective molar ratios of PNVCL and PVAc blocks in the star copolymer; the dn/dc value for PNVCL is 0.109 mL/g [35] and the dn/dc value for PVAc is 0.054 mL/g [65]. ^f^ Determined by ^1^H NMR in CDCl_3_.

**Table 3:** Characteristics of the star ((PNVCL-*co*-PNVP)-*b*-PVAc)_6_ copolymers.

Sample	(PNVCL_0.83_-*co*-PNVP_0.17_)_6_^a^				((PNVCL-*co*-PNVP)-*b*-PVAc)_6_				
	M_n GPC_ (g/mol) ^b^	Ð ^b^	D_h_^c^ (nm)	LCST (°C) ^d^	M_n GPC_ (g/mol) ^e^	Ð ^e^	PVAc (mol %) ^f^	D_h_^c^ (nm)	LCST (°C) ^d^
((PNVCL-*co*-PNVP)-*b*-PVAc)_6_-1	28,000	1.01	8.5 (1.8)	41	37,460	1.1	13.5	8.4 (1.8)	27
((PNVCL-*co*-PNVP)-*b*-PVAc)_6_-2					33,800	1,1	10	9.1 (1.5)	34
((PNVCL-*co*-PNVP)-*b*-PVAc)_6_-3					51,930	1.1	48.0	*	*

^a^ The molar ratio in (PNVCL_0.83_-*co*-PNVP_0.17_)_6_ was determined by ^1^H NMR in CDCl_3_. ^b^ By GPC in THF at 35 °C with RI and LS detectors using polystyrene linear standards for calibration of the LS detector. The dn/dc value used was calculated by using the following formula according to the composition of the copolymer by ^1^H NMR: dn/dc = 0.109(X) + 0.1194(Y), where X and Y are the respective molar ratios of PNVCL and PNVCL block in the star copolymer; the dn/dc value for PNVCL is 0.109 mL/g [35] and 0.1194 mL/g [72] for PNVP. ^c^ Determined by DLS (1 mg/mL) in water at room temperature. ^d^ Determined by DLS (1 mg/mL) in water. ^e^ The dn/dc value used was calculated by using the following formula according to the composition of the copolymer by ^1^H NMR: dn/dc = 0.109 (X) + 0.054 (Y) + 0.1194 (Z) where X, Y and Z are the respective molar ratios of PNVCL and PVAc block in the star copolymer; the dn/dc value for PNVCL is 0.109 mL/g [35], 0.1194 mL/g [72] for PNVP and 0.054 mL/g [65] for PVAc. ^f^ Determined by ^1^H NMR in CDCl_3_. * The sample was totally insoluble in water.

**Table 4:** Synthetic conditions and characteristics of the star [PVAc-*b*-(PNVCL-*co*-PNVP)]_6_ block copolymers.

Entry	(PVAc)_6_-macroCTA M_n GPC_ (g/mol) ^a^	Copolymer ^b^	M_n GPC_ (g/mol) ^c^	Ð ^c^	PVAc:PNVCL:PNVP (mol %) ^d^	LCST (°C) ^e^	D_h_^f^ (nm)	D_AFM_^g^ (nm)
1	9700	(PVAc_22_-*b*-PNVCL_11_)_6_	17,420	1.2	11:89:0	24	240	388
2	11,980	(PVAc_30_-*b*-(PNVCL_28_-*co*-PNVP_17_))_6_	42,290	1.1	42:47.5:10.5	36	234	245
3	6600	(PVAc_17_-*b*-(PNVCL_10_-*co*-PNVP_7_))_6_	36,480	1.1	47:41.4:11.6	40	115	99

^a^ By GPC in THF at 35 °C with RI and LS detectors using polystyrene linear standards for calibration of the LS detector. The dn/dc value for PVAc is 0.054 mL/g [65]. ^b^ The subscript numbers represent the repeating units of each PVAc, PNVCL or PNVP blocks estimated by using M_n_ GPC values. ^c^ The dn/dc value used was calculated by using the following formula according to the composition of the copolymer by ^1^H NMR: dn/dc = 0.109 (X) + 0.054 (Y) + 0.1194 (Z) where X, Y and Z are the respective molar ratios of PNVCL, PVAc and PNVP block in the star copolymer; the dn/dc value for PNVCL is 0.109 mL/g [35] the dn/dc value for PVAc is 0.054 mL/g [65] the dn/dc value for PNVP is 0.1194 mL/g [72]. ^d^ Determined by ^1^H NMR in CDCl_3_. ^e^ Determined by DLS, (1 mg/mL) in water. ^f^ Determined by DLS, (1 mg/mL) in water at room temperature. ^g^ Determined by AFM (atomic force microscopy) over mica substrates using the intermittent contact mode. D_AFM_ (diameter calculated by atomic force microscopy).

**Table 5:** Properties of MTX-loaded star polymer aggregates.

Sample	D_h_ (nm) ^a^	LCST (°C) ^b^	LC (%) ^c^	EE (%) ^d^	Cumulative Drug Release (%)		
					33 °C	37 °C	40 °C
					24 h		
(PVAc_30_-*b*-(PNVCL_28_-*co*-PNVP_17_))_6_	244	36	4.3	40	49	35	32
(PVAc_17_-*b*-(PNVCL_10_-*co*-PNVP_7_))_6_	123	40	7.0	66	33	41	40

^a^ Determined by DLS (1 mg/mL); the aggregates were prepared by direct dispersion in water. ^b^ Determined in water by DLS (1 mg/mL). ^c^ (LC) Loading content %. ^d^ (EE) Encapsulation efficiency %.

## Supplementary Materials

The following are available online at www.mdpi.com/2073-4360/10/1/20/s1, Figure S1: ^1^H NMR spectrum (400 MHz) of hexafunctional bromide RAFT agent precursor, Figure S2: ^13^C NMR spectrum (100 MHz) of hexafunctional bromide RAFT agent precursor, Figure S3: ^1^H NMR spectrum (400 MHz) of hexafunctional RAFT agent in CDCl_3_, Figure S4: ^13^C NMR (100 MHz) spectrum of hexafunctional RAFT agent in CDCl_3_, Figure S5: ^1^H NMR (400 MHz) spectrum of star (PNVCL)_6_ polymers in CDCl_3_, Figure S6: (**a**) UV−vis absorption spectra recorded for 0.24 mg/mL star (PNVCL)_6_ polymer solutions in THF before and after xanthate end-group removal ([ACVA]/[ (PNVCL)_6_] = 10.0, 80 °C, 24 h); (**b**) Normalized GPC traces in THF for star (PNVCL)_6_ polymers before and after treatment with ACVA, Figure S7. Normalized GPC (gel permeation chromatography) traces in THF (tetrahydrofuran) for star PNVCL polymers before and after treatment with ACVA, Figure S8: Normalized GPC traces in THF for star (PNVCL)_6_ polymers and star (PNVCL-*b*-PVAc)_6_ block copolymers, Figure S9: ^1^H NMR (400 MHz) spectrum of a star (PNVCL-*b*-PVAc)_6_ copolymer in D_2_O, Figure S10: Stability of aggregates from star (PNVCL-*b*-PVAc)_6_ block copolymers in aqueous solution (1 mg/mL); (a) Sample (PNVCL_99_-*b*-PVAc_21_)_6_ with 20 mol % of PVAc; (b) Sample (PNVCL_51_-*b*-PVAc_2_)_6_ with 5 mol % of PVAc, Figure S11: ^1^H NMR (400 MHz) spectrum of star (PNVCL-*co*-PNVP)_6_ copolymers M_n GPC_ = 28,000 g/mol, 17 mol % of PNVP, Figure S12: ^1^H NMR (400 MHz) spectrum of star [(PNVCL-*co*-PNVP)-*b*-PVAc]_6_ copolymers M_n GPC_ = 51,930 g/mol, 6% of PNVP, Figure S13: Normalized GPC traces in THF for star (PNVCL-*co*-PNVP)_6_ copolymers and star [(PNVCL-*co*-PNVP)-*b*-PVAc]_6_ block copolymers, Figure S14: ^1^H NMR (400 MHz) spectrum of star (PVAc)_6_ polymers (sample (PVAc_30_)_6_, Table S3, Entry 3, M_n NMR_ = 15,750 g/mol, M_n GPC_ = 11,980 g/mol, Ð = 1.2), Figure S15: Normalized GPC traces in THF for star (PVAc)_6_ polymers and the corresponding star block copolymer; (a) (PVAc_22_-*b*-PNVCL_11_)_6_ block copolymer with 89 mol % of PNVCL; (b) (PVAc_30_-*b*-(PNVCL_28_-*co*-PNVP_17_))_6_ block copolymer with 47.5 and 10.5 mol % of PNVCL and PNVP respectively, Figure S16: ^1^H NMR (400 MHz) spectrum in D_2_O of star (PVAc-*b*-(PNVCL-*co*-PNVP))_6_ block copolymers. Sample [PVAc_30_-*b*-(PNVCL_28_-*co*-PNVP_17_)]_6_, M_n GPC_ = 42,290 g/mol, Ð = 1.1 (Table 4, Entry 2), Figure S17: Drug release from unimolecular micelles of star (PNVCL-*b*-PVAc)_6_ block copolymers containing MTX at 37 °C, Table S1: Characteristics of star (PVAc)_6_ polymers, Table S2: Characteristics of star PNVCL polymers after treatment with ACVA, Table S3: Characteristics of star PNVCL polymers dispersed in water, ethanol and THF, Table S4: Characteristics of star PNVCL polymers dispersed in water, ethanol and THF, Table S5: Properties of MTX-loaded unimolecular micelles from star **(**PNVCL-*b*-PVAc)_6_ block copolymers.
