# Supplementary material for: Preparation of a Mini-Library of Thermo-Responsive Star (NVCL/NVP-VAc) Polymers with Tailored Properties Using a Hexafunctional Xanthate RAFT Agent
Source: Polymers (Basel). 2017 Dec 24;10(1):20. doi: 10.3390/polym10010020 (PMC6414999; doi:10.3390/polym10010020)
Supplement: Supplementary file 1 [file polymers-10-00020-s001.docx]

Supplementary Materials

**Preparation of a Mini-Library of Thermo-Responsive Star (NVCL/NVP-VAc) Polymers with Tailored Properties Using a Hexafunctional Xanthate
RAFT Agent**

Norma Aidé Cortez-Lemus and Angel Licea-Claverie

**
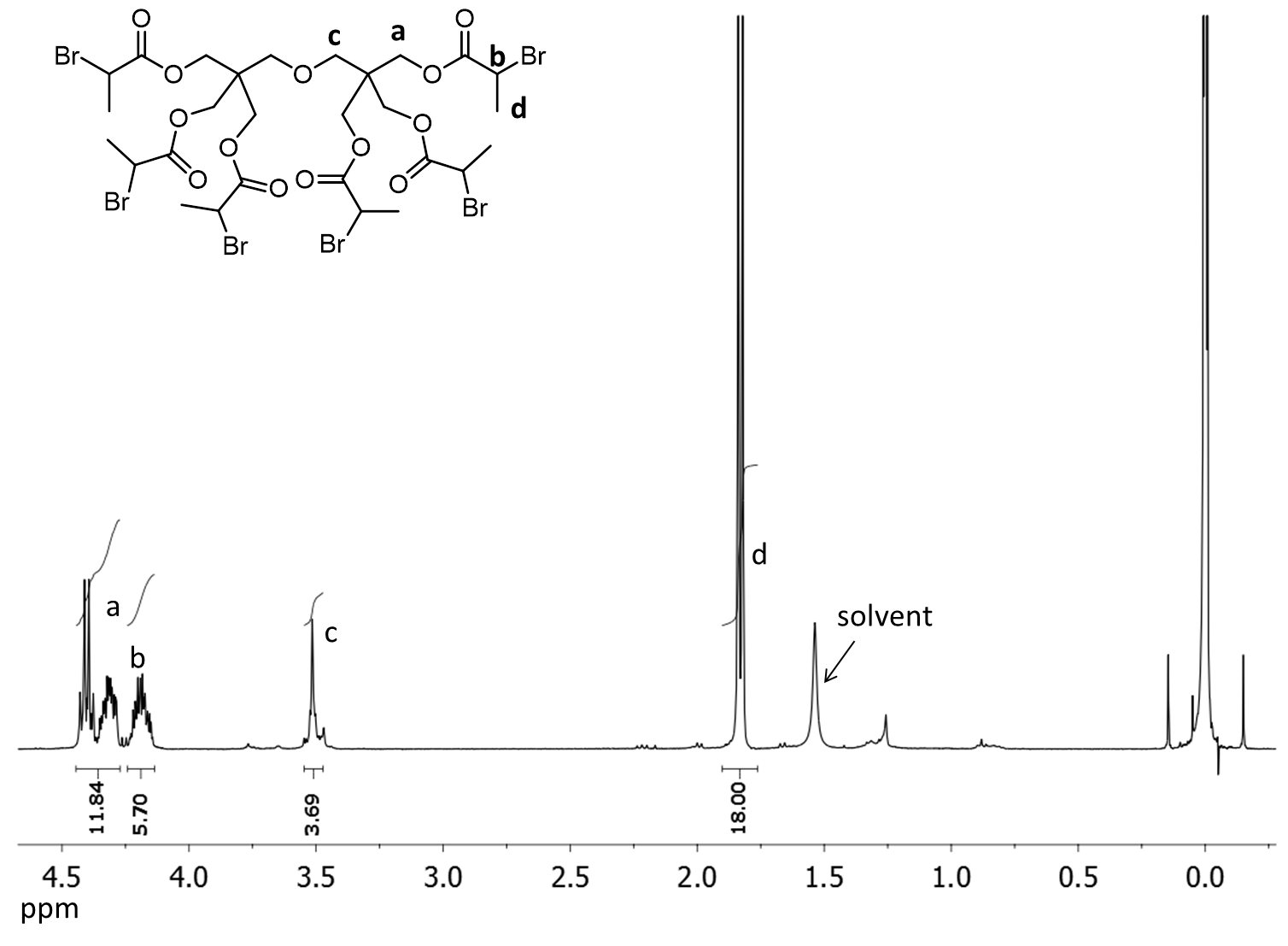
**

**Figure S1.** ^1^H NMR spectrum (400 MHz) of hexafunctional bromide reversible addition–fragmentation chain transfer (RAFT) agent precursor.

**
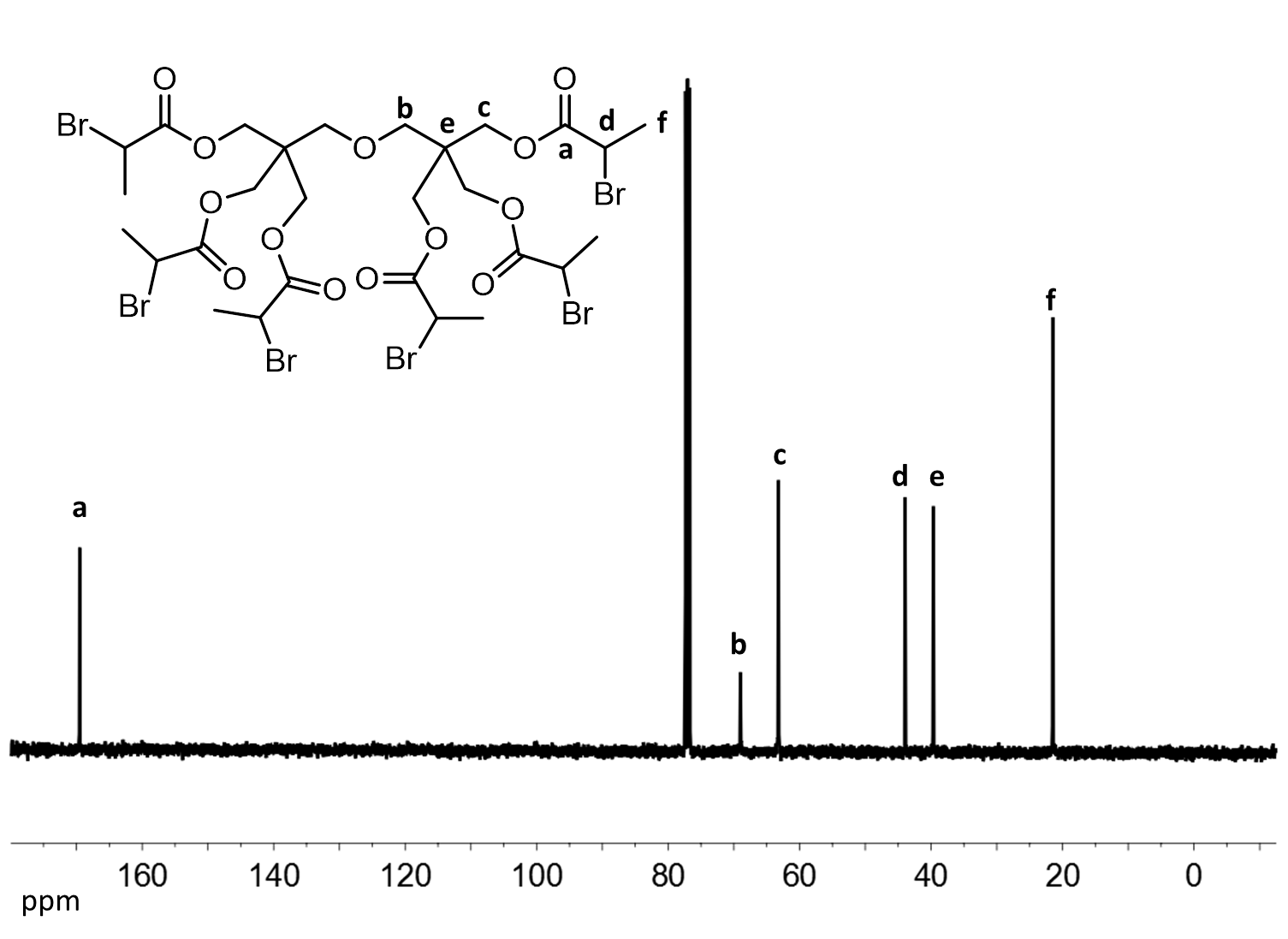
**

**Figure S2.** ^13^C NMR spectrum (100 MHz) of hexafunctional bromide RAFT agent precursor.

**
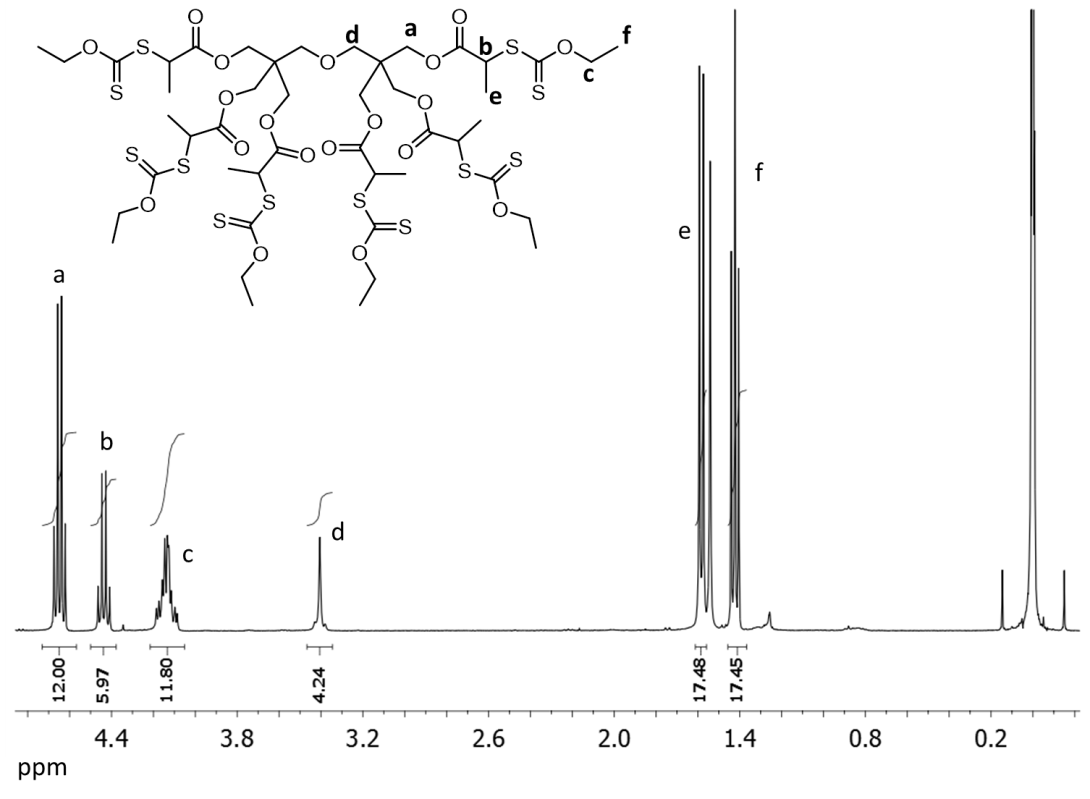
**

**Figure S3.** ^1^H NMR spectrum (400 MHz) of hexafunctional RAFT agent in CDCl_3_.


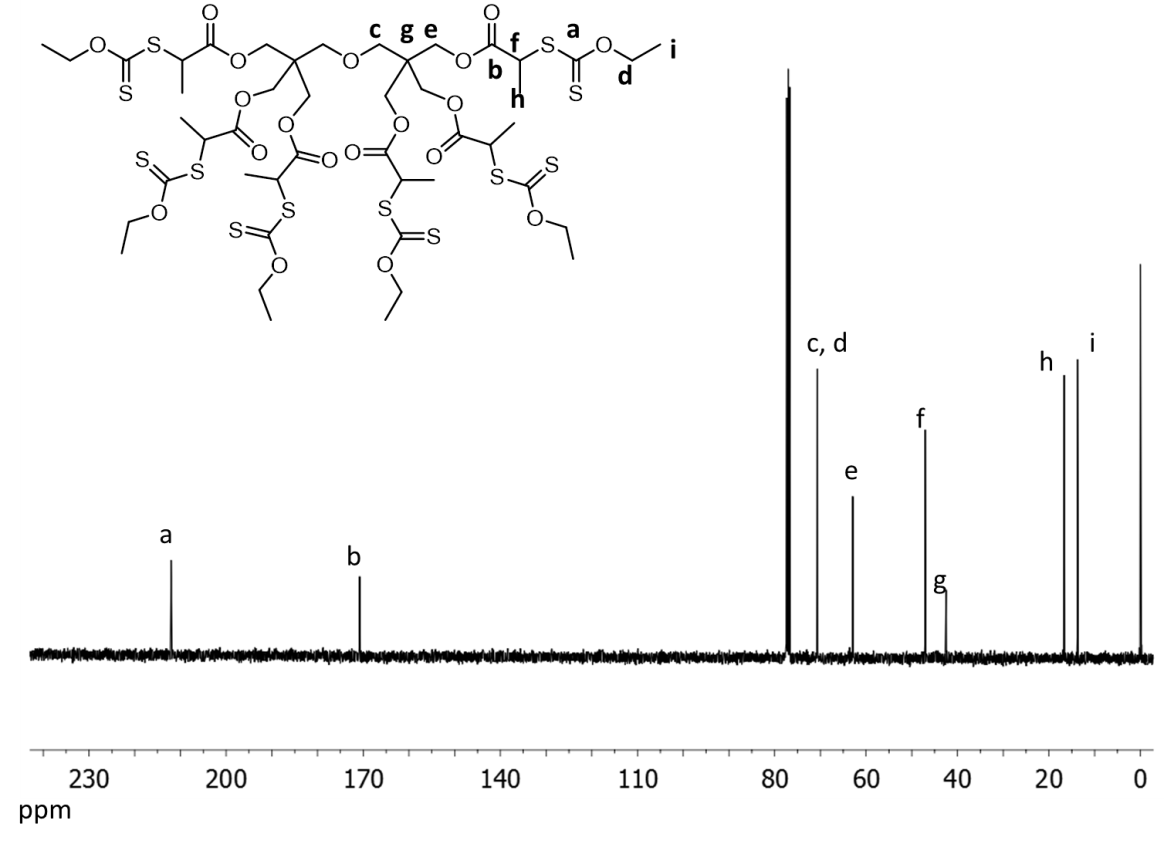


**Figure S4.** ^13^C-NMR (100 MHz) spectrum of hexafunctional RAFT agent in CDCl_3_.


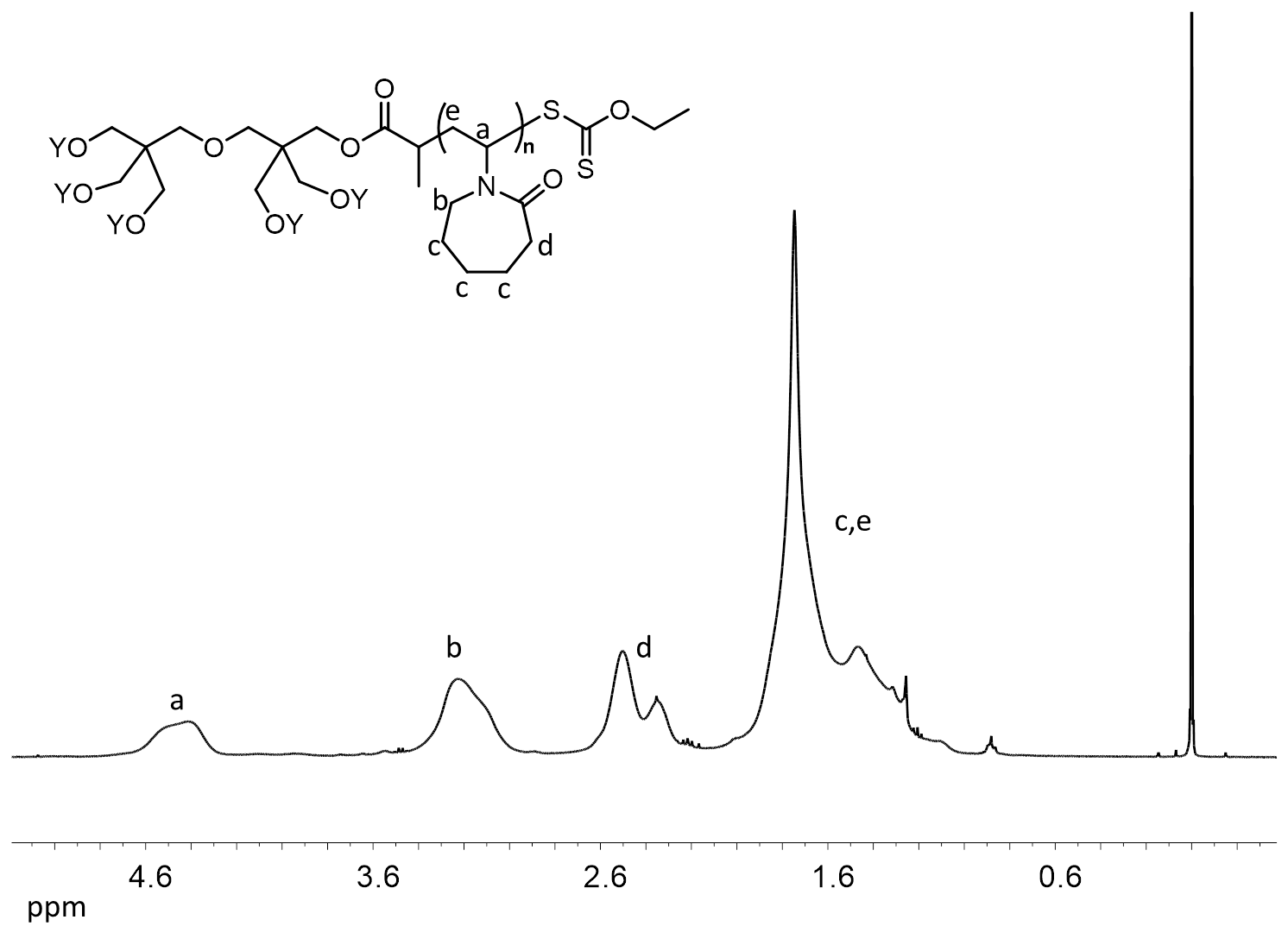


**Figure S5.** ^1^H-NMR (400 MHz) spectrum of star (PNVCL)_6_ polymers in CDCl_3_.

**Table S1.** Characteristics of star (PVAc)_6_ polymers.

| Entry | Sample ^a^ | [VAc]_o_:[CTA]_o_ | Yield (%) ^b^ | M_n GPC_ (g/mol) ^c^ | Ð ^c^ | M_n NMR_ (g/mol) ^d^ |
| --- | --- | --- | --- | --- | --- | --- |
| 1 | (PVAc_17_)_6_ | 270 | 40 | 6600 | 1.22 | 8750 |
| 2 | (PVAc_22_)_6_ | 500 | 33 | 9700 | 1.20 | 11,240 |
| 3 | (PVAc_30_)_6_ | 270 | 75 | 11,980 | 1.20 | 15,750 |

^a^ The subscript numbers represent the repeating units of PVAc estimated by using ^1^H NMR. ^b^ Determined gravimetrically. ^c^ By GPC in THF at 35 °C with RI detector using polystyrene linear standards for calibration. The dn/dc value for PVAc is 0.054 mL/g [1]. ^d^ Determined by ^1^H NMR CDCl_3_.

**Table S2.** Characteristics of star PNVCL polymers after treatment with ACVA.

|  | | before ACVA | | | after ACVA | | |
| --- | --- | --- | --- | --- | --- | --- | --- |
| Entry | Sample | M_n GPC_ (g/mol) ^a^ | LCST (°C) ^b^ | D_h water_ (nm) ^c^ | M_n GPC_ (g/mol) ^a^ | LCST (°C) ^b^ | D_h water_ (nm) ^c^ |
| 1 | (PNVCL_106_)_6_ | 88,580 | 33 | 13 (2.4) | 82,300 | 33 | 13.7 (2.7) |
| 2 | (PNVCL_52_)_6_ | 43,100 | 35 | 9.6 (1.9) | 39,300 | 35 | 10.5 (1.8) |
| 3 | (PNVCL_14_)_6_ | 11,880 | 36 | 6.6 (1.6) | 8900 | 39 | 7.9 (1.7) |
| 4 | (PNVCL_4_)_6_ | 2800 | * | * | 1800 | 31 | 21.6 (4.3) |

^a^ By GPC in THF with RI and LS detectors using a polystyrene linear standard for calibration of LS detector. ^b^ Determined by DLS in water (1 mg/mL). ^c^ D_h_ by volume (100% in all the samples) determined in water (1 mg/mL) by DLS analysis at 25 °C. Standard deviations in parenthesis; * The sample was insoluble in water.

|  |  |
| --- | --- |
| (a) | (b) |

**Figure S6.** (**a**) UV-vis absorption spectra recorded for 0.24 mg/mL star (PNVCL)_6_ polymer solutions in THF before and after xanthate end-group removal ([ACVA]/[(PNVCL)_6_] = 10.0, 80 °C, 24 h); (**b**) Normalized GPC traces in THF for star (PNVCL)_6_ polymers before and after treatment with ACVA.





**Figure S7.** Normalized GPC (gel permeation chromatography) traces in THF (tetrahydrofuran) for star PNVCL polymers before and after treatment with ACVA.

**Table S3.** Characteristics of star PNVCL polymers dispersed in water, ethanol and THF.

| Entry | Sample | M_n GPC_ (g/mol) ^a^ | LCST (°C) ^b^ | D_h water_ (nm) ^c^ | D_h EtOH_ (nm) ^c^ | D_h THF_ (nm) ^c^ | h (nm) ^d^ |
| --- | --- | --- | --- | --- | --- | --- | --- |
| 1 | (PNVCL_184_)_6_ | 153,400 | 33 | 19 (5.7) | 12 (3.4) | 4.7 (1.2) | 19.1 |
| 2 | (PNVCL_151_)_6_ | 125,800 | 33 | 17.5 (3.6) | 11.3 (2.9) | 4.2 (1.2) | 18.0 |
| 3 | (PNVCL_106_)_6_ | 88,580 | 33 | 13 (2.4) | 8.8 (2.3) | 3.5 (0.74) | 14.0 |
| 4 | (PNVCL_52_)_6_ | 43,100 | 35 | 9.6 (1.9) | 7.1 (1.4) | 3.1 (0.5) | 11.3 |
| 5 | (PNVCL_34_)_6_ | 28,100 | 37 | 7.9 (1.1) | 5.1 (1.1) | 2,6 (0.6) | 8.1 |
| 6 | (PNVCL_23_)_6_ | 19,100 | 38 | 7.1 (1.1) | 4.7 (0.88) | 5.2 (1.1) | 7.5 |
| 7 | (PNVCL_18_)_6_ | 15,000 | 38 | 5.7 (1.9) | 4.8 (1.1) | 2.02 (0.4) | 7.6 |
| 8 | (PNVCL_15_)_6_ | 12,800 | 36 | 5.4 (0.83) | 5.10 (1.3) | 3.1 (0.5) | 8.1 |
| 9 | (PNVCL_14_)_6_ | 11,880 | 36 | 6.6 (1.6) | 3.5 (0.74) | 1.5 (0.4) | 5.6 |
| 10 | (PNVCL_4_)_6_ | 3200 | * | * | 3.5 (0.73) | 4.04 (0.6) | 5.6 |

^a^ By GPC in THF at 35 °C with RI detector using polystyrene linear standards for calibration. The dn/dc value for PNVCL is 0.109 mL/g [2]. ^b^ Determined by DLS (detection angle = 90°) at 1 mg/mL. ^c^ D_h_ by volume (100% in all the samples) determined by DLS analysis at 25 °C. Standard deviations in parenthesis. ^d^ The end to end distance of a coiled polymer chain in a good solvent (h) [3] is calculated using following equation [3]: $\langle h\rangle^{2}=(2+\varepsilon)(3+\varepsilon)\langle R_{g}\rangle^{2}$ Where: $\varepsilon=2\nu-1$ and $\nu=0.6$ for good solvent (Flory), Taking R_g_ = R_h_ × 1.2 [4] * The sample was insoluble in water.





**Figure S8.** Normalized GPC traces in THF for star (PNVCL)_6_ polymers and star (PNVCL-*b*-PVAc)_6_ block copolymers.


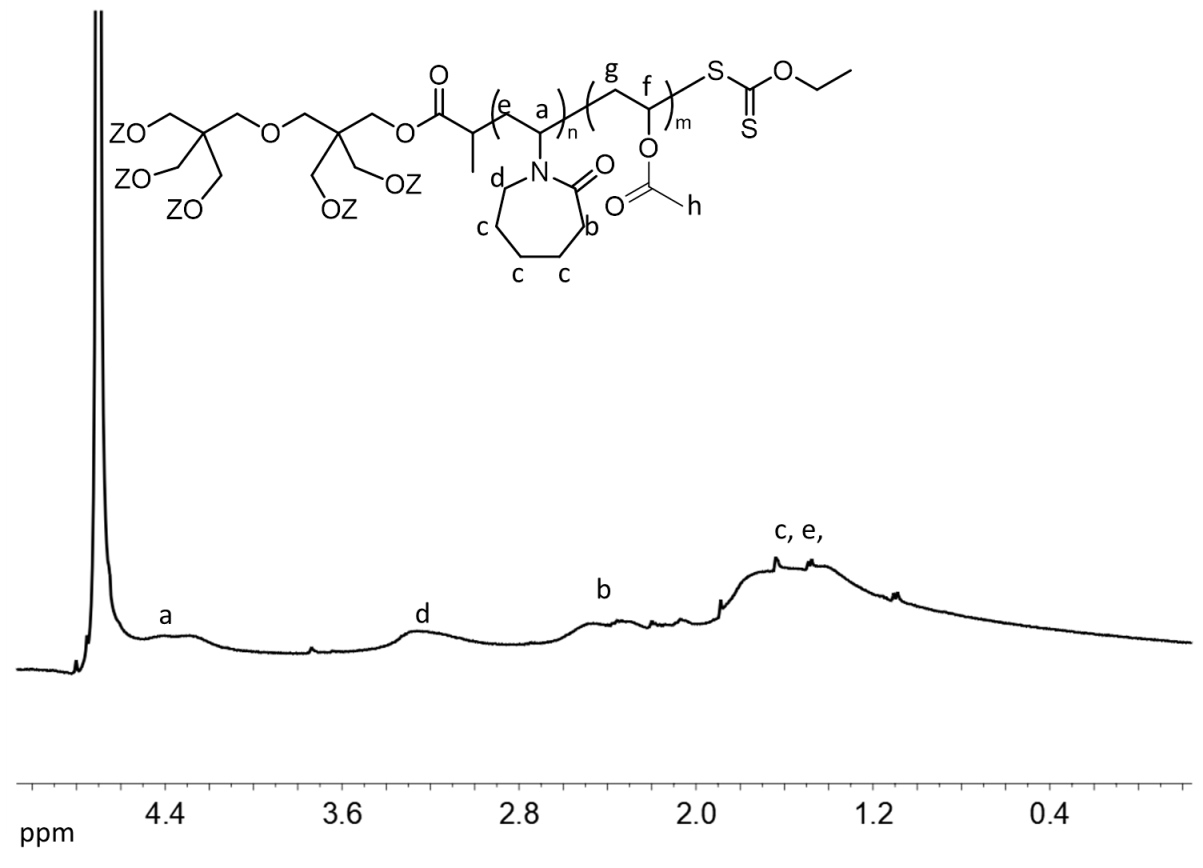


**Figure S9.** ^1^H-NMR (400 MHz) spectrum of a star (PNVCL-*b*-PVAc)_6_ copolymer in D_2_O.

|  |  |
| --- | --- |
| (a) | (b) |

**Figure S10.** Stability of aggregates from star (PNVCL-*b*-PVAc)_6_ block copolymers in aqueous solution (1 mg/mL); (a) Sample (PNVCL_99_-*b*-PVAc_21_)_6_ with 20 mol % of PVAc; (b) Sample (PNVCL_51_-*b*-PVAc_2_)_6_ with 5 mol % of PVAc .


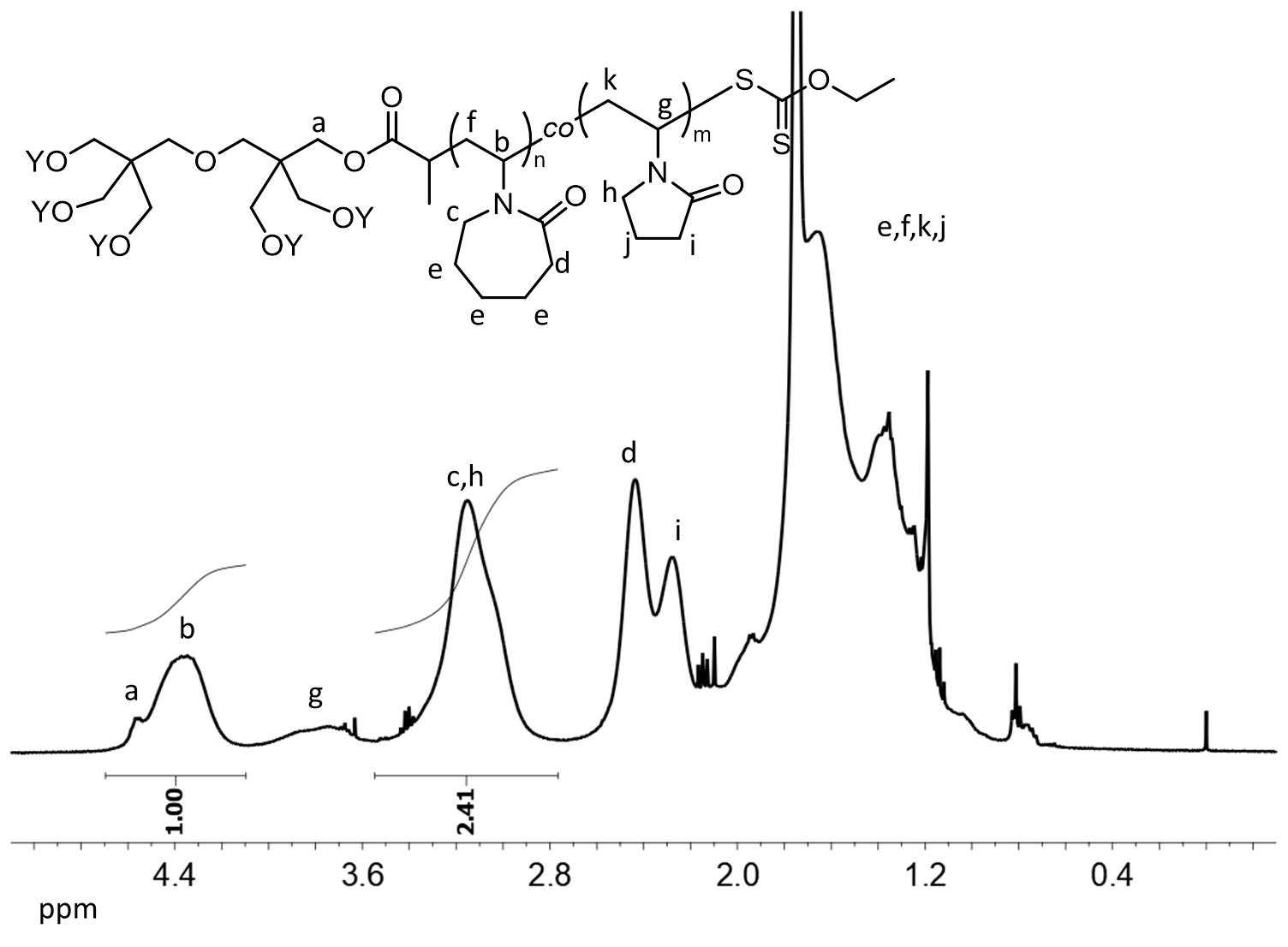


**Figure S11.** ^1^H NMR (400 MHz) spectrum of star (PNVCL-*co*-PNVP)_6_ copolymers M_n_ _GPC_ = 28,000 g/mol, 17 mol % of PNVP.


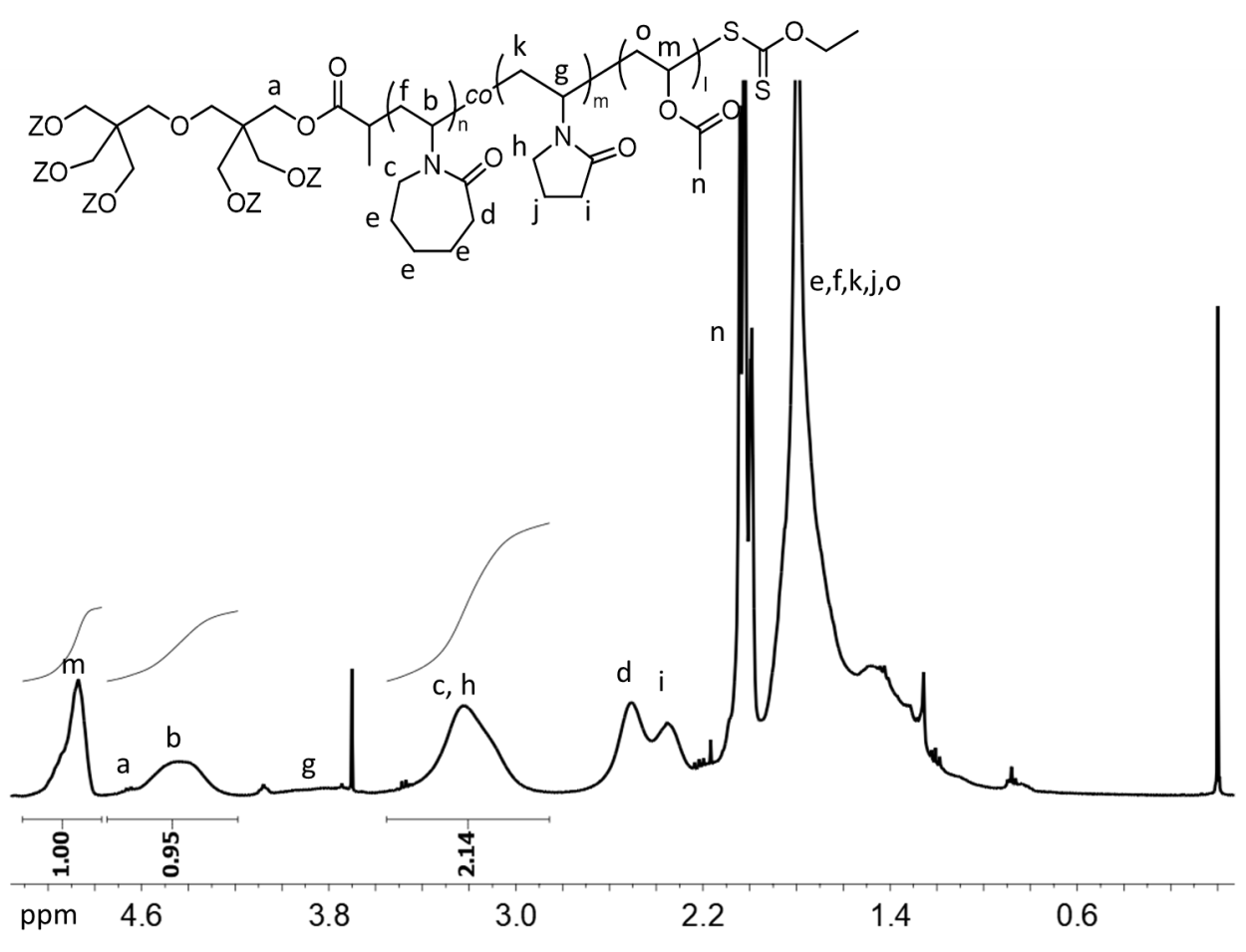


**Figure S12.** ^1^H NMR (400 MHz) spectrum of star [(PNVCL-*co*-PNVP)-*b*-PVAc]_6_ copolymers M_n_ _GPC_ = 51,930 g/mol, 6% of PNVP.

**

**

**Figure S13.** Normalized GPC traces in THF for star (PNVCL-*co*-PNVP)_6_ copolymers and star [(PNVCL-*co*-PNVP)-*b*-PVAc]_6_ block copolymers.


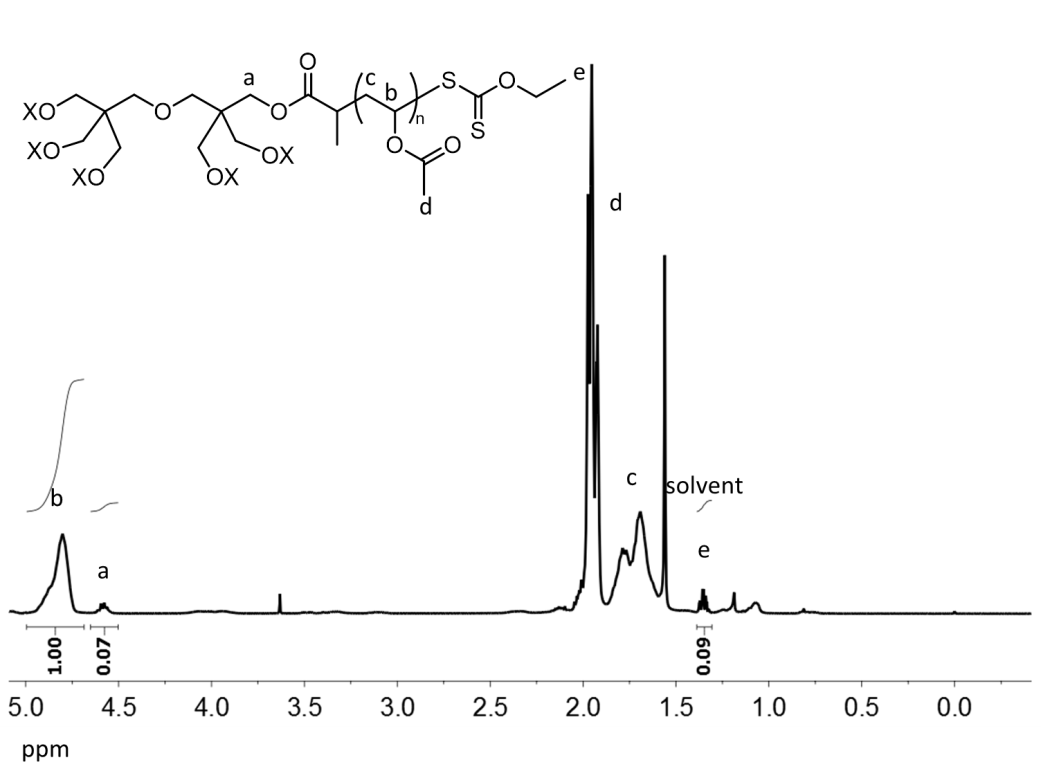


**Figure S14.** ^1^H NMR (400 MHz) spectrum of star (PVAc)_6_ polymers (sample (PVAc_30_)_6_, Table S3, entry 3, M_n NMR_ = 15,750 g/mol, M_n GPC_ = 11,980 g/mol, Ð = 1.2).

|  |  |
| --- | --- |
| (a) | (b) |

**Figure S15.** Normalized GPC traces in THF for star (PVAc)_6_ polymers and the corresponding star block copolymer; (a) (PVAc_22_-*b*-PNVCL_11_)_6_ block copolymer with 89 mol % of PNVCL; (b) (PVAc_30_-*b*-(PNVCL_28_-*co*-PNVP_17_))_6_ block copolymer with 47.5 and 10.5 mol % of PNVCL and PNVP respectively .


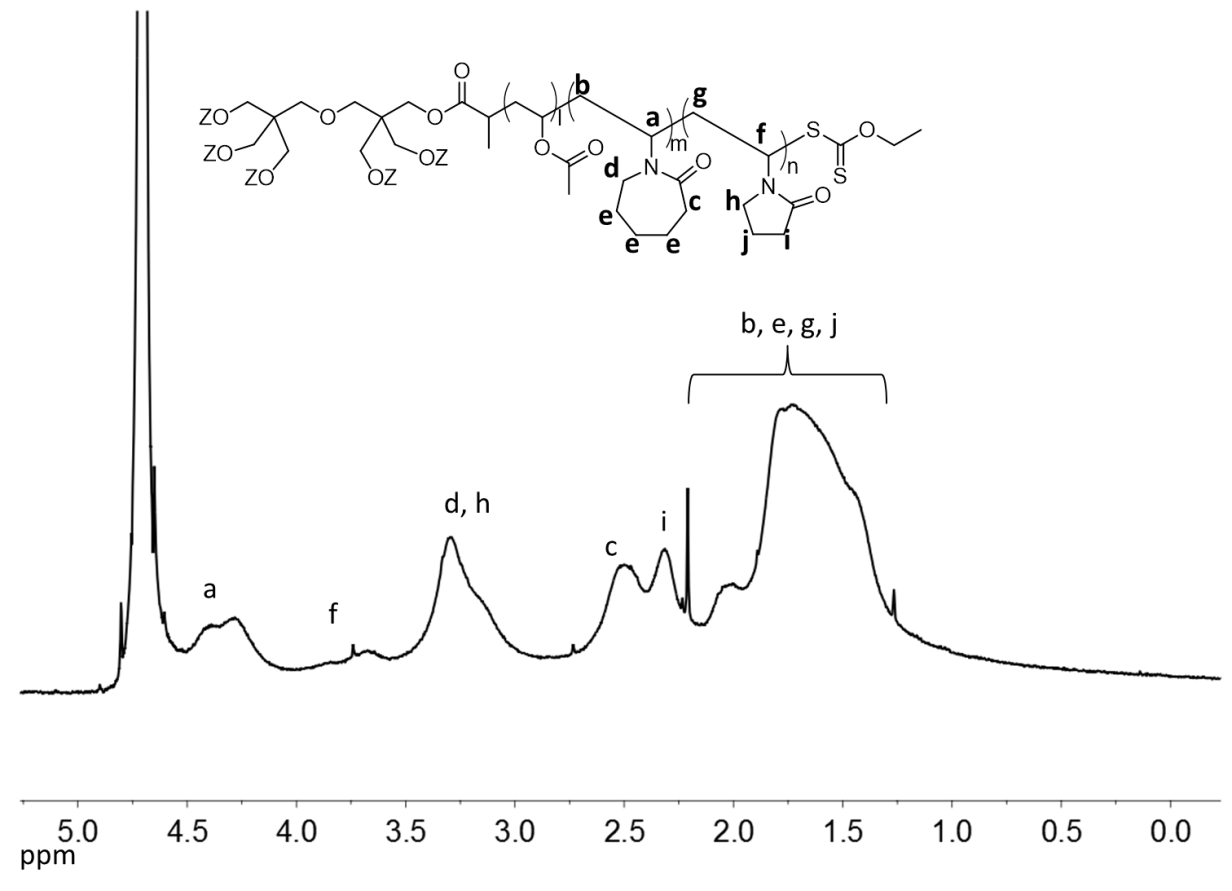


**Figure S16.** ^1^H NMR (400 MHz) spectrum in D_2_O of star (PVAc-*b*-(PNVCL-*co*-PNVP))_6_ block copolymers. Sample [PVAc_30_-*b*-(PNVCL_28_-*co*-PNVP_17_)]_6_, M_n GPC_ = 42,290 g/mol, Ð = 1.1
(Table 4, entry 2).

**Table S4.** Characteristics of star PNVCL polymers dispersed in water, ethanol and THF.

| Entry | Sample ^a^ | M_n GPC_ (g/mol) ^b^ | LCST (°C) ^c^ | D_h water_ (nm) ^d^ | D_h EtOH_ (nm) ^d^ | D_h THF_ (nm) ^d^ | h (nm) ^e^ |
| --- | --- | --- | --- | --- | --- | --- | --- |
| 1 | (PVAc_22_-*b*-PNVCL_11_)_6_ | 17,420 | 24 | 240 (83) | 339 (133) | 15.0 (2.7) | 23.9 |
| 2 | (PVAc_30_-*b*-(PNVCL_28_-*co*-PNVP_17_))_6_ | 42,290 | 36 | 234 (90) | 6.2 (1.4) | 11.5 (2.1) | 18.3 |
| 3 | (PVAc_17_-*b*-(PNVCL_10_-*co*-PNVP_7_))_6_ | 36,480 | 40 | 115 (61.9) | 4.8 (1.0) | 13.3 (2.4) | 21.2 |

^a^ The subscript numbers represent the repeating units of each PVAc, PNVCL or PNVP blocks estimated by using M_n_ GPC values. ^b^ By GPC in THF at 35 °C with RI and LS detector using a polystyrene linear standard for calibration of the LS detector. The dn/dc value used was calculated by using the following formula according to the composition of the copolymer by ^1^H NMR: dn/dc = 0.109 (X) + 0.054 (Y) + 0.1194 (Z) where X, Y and Z are the respective molar ratios of PNVCL, PVAc and PNVP block in the star copolymer; the dn/dc value for PNVCL is 0.109 mL/g [2], the dn/dc value for PVAc is 0.054 mL/g [1], the dn/dc value for PNVP is 0.1194 mL/g [5]. ^c^ Determined by DLS (detection angle = 90°) at 1 mg/mL. ^d^ D_h_ by volume (100% in all the samples) determined by DLS analysis at 25 °C. Standard deviations in parenthesis. ^e^ End to end distance (h) [3] of coiled star in a good solvent (THF) taking R_g_ = R_h_ × 1.2 according to Burchard [4] as average value for stars with monodisperse arms.

**Table S5.** Properties of MTX-loaded unimolecular micelles from star **(**PNVCL-*b*-PVAc)_6_ block copolymers.

|  | | | | | Cumulative Drug Release (%) |
| --- | --- | --- | --- | --- | --- |
|  |  |  |  |  | 33 °C |
| Sample | D_h_ (nm) ^a^ | LCST (°C) ^b^ | LC (%) ^c^ | EE (%) ^d^ | 24 h |
| (PNVCL_99_-*b*-PVAc_21_)_6_ | 13.3 (3.6) | 28 | 4 | 37 | 81.4 |
| (PNVCL_51_-*b*-PVAc_2_)_6_ | 9 (2.3) | 27 | 5 | 48 | 54.0 |
| (PNVCL_99_-*b*-PVAc_18_)_6_ | 13.3 (3.6) | 29 | 4 | 35 | 70.3 |

^a^ Determined by DLS (1 mg/mL) in water at 20 °C. ^b^ Determined by DLS (1 mg/mL) in water. ^c^ (LC) Loading content %. ^d^ (EE) Encapsulation efficiency %.





**Figure S17.** Drug release from unimolecular micelles of star (PNVCL-*b*-PVAc)_6_ block copolymers containing MTX at 37 °C.

References

1. Brandrup, J.; Immergut, E.H.; Grulke, E.A. *Polymer Handbook*, 4th ed.; Wiley-Interscience: New York, NY, USA, 1999.
2. Cortez-Lemus, N.A.; Licea-Claverie, A. Poly(*N*-vinylcaprolactam), a comprehensive review on a thermoresponsive polymer becoming popular. *Prog. Polym. Sci.* **2016**, *53*, 1–51.
3. Arndt, K.F.; Müller, G. *Polymercharakterisierung*; Carl Hanser Verlag: München, Germany, 1996.
4. Burchard, W. Solution properties of branched macromolecules. *Adv. Polym. Sci*. **1999**, *143*, 113–194.
5. Fandrich, N.; Falkenhagen, J.; Weidner, S.M.; Pfeifer, D.; Staal, B.; Thünemann, A.F.; Laschewsky, A. Characterization of new amphiphilic block copolymers of *N*‐vinyl Pyrrolidone and vinyl acetate, 1—Analysis of copolymer composition, end groups, molar masses and molar mass distributions. *Macromol.* *Chem. Phys.* **2010**, *211*, 869–878.

© 2017 by the authors; licensee MDPI, Basel, Switzerland. This article is an open access article distributed under the terms and conditions of the Creative Commons Attribution (CC BY) license (http://creativecommons.org/licenses/by/4.0/).
